# Modelling and simulation of electrochemical processing of solid polyolefin wastes by non-thermal plasma treatment: a mini-review

**DOI:** 10.3389/fchem.2025.1615725

**Published:** 2025-12-05

**Authors:** Mohammad Jakir Hossain Khan, Jochen Uebe, Zilvinas Kryzevicius, Audrius Senulis, Audrone Zukauskaite

**Affiliations:** Engineering Department, Faculty of Marine Technology and Natural Sciences, Klaipeda University, Klaipeda, Lithuania

**Keywords:** model development, non-thermal plasma, polyolefin wastes, reactor simulation, reaction engineering

## Abstract

Creating accurate models to explain the reaction mechanisms in thermochemical processing of solid polyolefins and their derivatives using non-thermal plasma (NTP) technology is crucial for improving recycling and reuse efforts. This area has gained significant attention over the past few decades. The model for polyolefin breakdown involves a mix of complex free radical reactions, along with formal and molecular processes. NTP reactors provide an environment with enhanced reactivity and performance, making them highly efficient for treating solid polyolefins and ideal for producing clean energy and other valuable products from polyolefin waste. Therefore, developing adaptable and precise simulations to identify the best geometric configurations for NTP reactors is key to improving their performance. Utilising various computational techniques and integrating suitable algorithms to build models that meet design goals and predict results offers a cutting-edge approach for engineering applications. Mathematical modelling and cutting-edge computational simulations can enhance themselves by incorporating data and verifying results experimentally, with a focus on linking inputs to anticipated results. This method is crucial in interpreting the mathematical connections among various intricate procedures and actual response circumstances. In this study, a concise overview of new, promising research advances in the treatment of polyolefin waste using NTP has been presented. The subjects covered in this study include i) advancements in various class modelling techniques for analysing and understanding the reaction dynamics of NTP-treated polyolefin wastes, ii) simulation approaches for NTP reactors, and iii) existing challenges and future outlooks. The process can be commercialised due to the potentially high market value of its products, which include chemicals and fuels. Additionally, by creating appropriate models through solving sets of equations and assessing system performances under the complex conditions required for these products, the selectivity of this technology can be enhanced. An immediate requirement exists to summarise the current methods, pinpoint the technological limitations, and outline necessary research in this developing area.

## 1 Introduction

The valorisation of polyolefin-derived wastes, by generating economic benefits while minimising environmental harm, plays a crucial role in sustainable development. Plastic waste from polyolefins is projected to reach 25 gigatonnes globally by 2050 (Geyer et al., 2017). This enormous waste stream presents substantial opportunities, especially in the production of renewable fuels by utilising an optimally structured reaction framework. In this case, using non-thermal plasma systems powered by renewable electricity could effectively convert waste plastics into valuable energy sources, addressing both waste management and energy needs simultaneously. The elaborate investigation into the non-thermal plasma (NTP) systems can enhance the efficiency and sustainability of fuel production by addressing multiple engineering challenges. NTP technologies are rapidly gaining global attention because of their unique ability to operate at atmospheric pressure and ambient temperatures. Of late, cold atmospheric plasma systems have shown promising applications across various industries, including plastic waste treatment (Khan et al., 2024a), biomedical applications (Khan et al., 2020), food industry starch modification (Chankuson et al., 2023), and low-temperature catalysis in environmental sciences (Song et al., 2024). NTP is produced in a reactor containing the gas to be processed, where a potential difference applied between two electrodes creates an electric field (Mei and Tu, 2017). This accelerates electrons, triggering collisions with molecules that result in ionisation, excitation, and dissociation. These collisions promote reactions between different species and break down molecules inside the reactor.

At the same time, the field of plasma modelling has advanced from a simple charged ideal gas model to various modelling techniques such as complex fluid and wave plasma models (Haas, 2019; Mei and Tu, 2017). In a study, scientists utilised a modelling method to depict NTP as a sequence of reaction kinetics, highlighting the chemical reactions and their characteristics in the NTP system (Maitre et al., 2020). The computation of reaction rates under NTP conditions involves intricate analysis of electron dynamics, chemical kinetics, and excited state properties. The precision of these models highly depends on the selection of appropriate model equations for calculating rate constants, often requiring complex coefficients integration and empirical tunings. Poutsma et al. (2000) outlined the fundamental responses of free radicals involved in the pyrolysis process. They outlined the main link between thermochemical and kinetic elements to calculate rate constants when real-time data are not available. Models have been developed by examining the impacts of tuning reaction parameters for the degradation rates of polyolefins and to produce olefin monomers. It has been observed that tuning NTP electrical parameters during reactions can improve the alignment of model predicted outcomes with experimental data (Sundaram and Froment, 1978).

## 2 Review scope

Recycling plastic waste is considered the most eco-friendly approach. However, after multiple cycles of mechanical recycling, plastics suffer from diminished mechanical properties due to the shortening of polymer chains. To counteract this, additives are often introduced, but they can interfere with future recycling efforts. Longer stability and the provision of additional material properties are the two main expected aspects when incorporating additives into polyolefins during commercial-scale manufacturing. Stabilisers for higher temperatures, plasticisers, phosphoric acid, pentaerythritol, crosslink agents, etc., are commonly used as additives during olefin polymerisation. Every additive plays a diverse role in providing and improving the end-product functional characteristics of a polyolefin product. These additives show strong persistence against thermochemical recycling processes and cause interferences by inducing side reactions (Millican and Agarwal, 2021). Consequently, incineration with energy recovery has been promoted as an alternative for plastic waste management as it reduces waste volume to less than 10% of its original size while recovering energy (Neuwahl et al., 2019). The commonly used polyolefin (soft and hard plastics) are particularly suited for this method due to their low moisture content and high calorific value (Kumar and Samadder, 2017). According to the literature, the calorific value of various plastics ranges from 43 to 51 MJ/kg (polyethylene, 43 MJ/kg, and mixed plastics, 30–40 MJ/kg), which is very close to fuels (methane, 53 MJ/kg; gasoline, 46 MJ/kg; and fuel oil, 43 MJ/kg) (Jaafar et al., 2022; Panda et al., 2010). Notably, incineration also results in significant CO_2_ emissions and air pollutants such as dioxins, NOx, and SOx, which increase the operational costs of flue gas treatment systems (Mukherjee et al., 2020). Thus, alternative technologies such as biochemical conversion, pyrolysis, and gasification have been explored (Dai et al., 2023; Lee et al., 2023; Arena, 2012; Zhao et al., 2022). Yet, challenges remain with scalability and emissions, and without C capture and storage, the emission levels of plastic waste-to-energy processes remain similar to those of fossil fuel power plants.

Plasma technology has emerged as a promising valorisation technique due to its high product yields. As a gasification method, plasma processing uses a plasma source, such as a plasma torch, to provide external heat, making it easily controllable and capable of operating at high temperatures over short durations (Sajid et al., 2022). Compared to incineration, plasma treatment has been shown to have a more favourable environmental impact in terms of greenhouse gas (GHG) emissions and ozone depletion. However, further assessments are needed to confirm its overall environmental benefits (Shao et al., 2022).

The literature indicates that in contrast to plasma processing the incineration results in significant environmental hazards, particularly concerning dioxin emissions, greenhouse gases (GHGs), and acid gases, as delineated in Table 1. 

**TABLE 1:** Calculated pollution factors by technology (Tang et al., 2020).

Pollutant	Incineration	Plasma gasification
CO_2_ (kg)	331.3	331.4
CO (kg) 0.4 0.2	0.4	0.2
SO_2_ (kg)	0.4	0.1
HCl (kg)	0.3	0.1
Dioxins (kg I-TEQ)	5.1 × 10^−7^	2.5 × 10^−7^

Table 1 clearly illustrates that the plasma technique significantly contributes to reducing GHG emissions compared to incineration. The hybridised environmental impact assessment (EIA) model is useful for calculating GHG generation during energy production from plastic wastes by applying incineration and plasma techniques (Tang et al., 2020).

Two plasma methods, namely, thermal plasma (TP) and NTP, are being evaluated for polyolefin waste treatment. Unlike TP, NTP promotes reforming reactions by bypassing traditional pathways and lowering the energy barriers of reactions using radicals, all without significant temperature increases (Ramos and Rouboa, 2022). Both TP and NTP rely on electromagnetic fields to accelerate electrons, but TP features higher electron densities, leading to more collisions between molecules and electrons, while NTP operates with lower electron densities and fewer collisions (Petitpas et al., 2007). As a result, NTP achieves high vibrational and electronic temperatures of 1–10 eV, while the gas temperature stays relatively low, at approximately 20 °C–100 °C. In contrast, TP’s gas temperature matches its electron temperature. Additionally, NTP consumes less energy and has lower maintenance costs compared to TP. NTP sources, such as dielectric barrier discharge (DBD), gliding arc discharge, and corona discharge, have been used to break down solid polymers and reform different fuels (Puliyalil et al., 2018).

In the 2022 Plasma Roadmap (Adamovich et al., 2022), process modelling, simulation, optimisation, and scaling up are the key technological hurdles that could impact chemical feedstock processing and hydrocarbon production. As stated in the 2022 Roadmap, enhancing and expanding the processes of various plasma science innovations—from laboratory experiments to industries and society—is vital for their success and practical implementation. A key objective moving forward is to create accurate and reliable computational models based on physics or chemistry for designing and optimising devices. This involves extensive research in selecting, developing, and validating models. Issues related to technical and physical aspects were identified in adapting and optimising plasma devices, such as designing small and miniaturised devices for plasma treatment and monitoring various plasma and target parameters during treatment. The objective is to formulate models that incorporate the capability to modulate plasma dynamics for designated petrochemical processes, while considering that the output generated is subject to ongoing assessment of both the desired reaction rates and the efficacy of the plasma.

Regrettably, a majority of the research papers concentrate on the observed effects of plasma-assisted valorisation (PAV), with little to no information provided about the specific chemical and physical processes of plasma enhancement and the efforts towards system analysis. Moreover, researchers have not assessed the potential, opportunities, and difficulties in utilising new computational advancements and tools to analyse the reaction pathways. They have also not summarised the indispensable kinetic equations and the related dynamic key factors impacting the conversion of polymeric waste into fuels. Proposing a brief literature review that blends up-to-date information on numerical and experimental approaches can help address these issues in the field of interest.

## 3 Models for non-thermal plasma processing of polyolefin waste

To enhance the clarity of this review, the subsequent sections will first discuss the developed computational fluid dynamics (CFD) simulations for non-thermal plasma reactors applied to discarded polyolefin. The discussion will then transition to reaction kinetics and the modelling of electrical parameters, followed by energy and exergy calculations essential for scaling NTP systems. The significance of statistical optimisation in determining process parameters for effective scaling will also be highlighted in this review. Moreover, the importance of simulating NTP systems and modelling waste polyolefin reactions will be emphasised before reaching the final conclusions. This structured approach will ensure a comprehensive understanding of the interplay between simulation, kinetics, and optimisation in enhancing NTP reactor performance. This methodical assessment aims to provide insights into the effectiveness of NTP systems while addressing the challenges associated with scaling up the technology.

### 3.1 Computational fluid dynamics simulations of non-thermal plasma reactors for polyolefin waste processing

CFD analysis is still in its early phases, particularly when applied to processing polyolefin-derived wastes in NTP-powered systems. While CFD has been widely used in chemical reactor studies, current simulations are often oversimplified and lack consideration for multiscale structures (e.g., assuming homogeneity in electrochemical dynamics, mass transfer, and reaction models and using 2D instead of 3D models). Advancing CFD to solve conservation and momentum equations in multiphase flows represents a cutting-edge research area that can visualise fundamental phenomena without the need for real-time experiments (El Sheikh et al., 2019; Khan et al., 2016a; Khan et al., 2014; Khan et al., 2016b; Kwon and Im, 2024).

In NTP reactors, CFD offers the added benefit of providing detailed insights into the distribution of gaseous products and how they transform based on the tuning of electrical power parameters such as voltage, current, and frequency (Khan et al., 2024a). This information is crucial as the reactants are often in the form of highly reactive ions during plasma treatment, leading to higher reaction yields. Even though experimental tuning of parameters in NTP reactors is still essential, the complex behaviour of reactive gases makes dynamic visualisation challenging.

The Euler–Euler multiphase mathematical model, used in CFD, is particularly effective for simulating NTP reactors, helping comprehend the intricate impacts of physical and chemical factors such as the steam-to-fuel ratio (SFR), equivalence ratio (ER), and plasma power input on the gasification of solid plastic waste in a fixed bed. This model can simulate temperature and velocity fields, gas and solid composition changes, and other electrochemical dynamics within the reactor (Atan et al., 2019). The next section outlines the key equations required for CFD simulations in NTP system development.

In the context of CFD, additional scalar transport equations might be required for modelling surface reactions enhanced by NTP, particularly in pyrolysis, combustion, and gasification processes. A key advantage of CFD is its ability to simulate both single-phase and multiphase electrochemical reaction dynamics within the NTP system.

For an arbitrary scalar $\phi_{k}$ , CFD suggests solving the equation

$$\frac{dp\phi_{k}}{dt}+\frac{d}{dx_{i}}\left(\rho u_{i}\phi_{k}-\Gamma_{k}\frac{d\phi_{k}}{dx_{i}}\right)=S_{\phi_{k}\;}k=1,\ldots ,N,$$ (1)

where $\Gamma_{k}$ and $S_{\phi_{k}\;}$ are, respectively, the diffusion coefficient and source term supplied for each of the *N* scalar equations. Note that $\Gamma_{k}$ is defined as a tensor in the case of anisotropic diffusivity. The diffusion term is thus

$$\nabla \cdot \left(\Gamma_{k}\cdot \phi_{k}\right).$$ (2)

For isotropic diffusivity, $\Gamma_{k}$ can be written as $\Gamma_{k}I$ , where *I* is the identity matrix.

For the steady-state case, it is highly recommended to solve one of the three following equations depending on the method used to compute the convective flux.

If convective flux is not to be computed, available software will solve the equation

$$-\frac{\partial }{{\partial x}_{i}}\left(\Gamma_{k}\frac{\partial \phi_{k}}{\partial x_{i}}\right)S_{\phi_{k}\;}k=1,\ldots ,N,$$ (3)

where $\Gamma_{k}$ and $S_{\phi_{k}\;}$ are, respectively, the diffusion coefficient and source term to be defined for each of the *N* scalar equations.

If convective flux is to be computed using the mass flow rate, it is recommended to solve the equation

$$\frac{\partial }{{\partial x}_{i}}\left({\rho u_{i}\phi_{k}-\Gamma }_{k}\frac{\partial \phi_{k}}{\partial x_{i}}\right)S_{\phi_{k}\;}k=1,\ldots ,N.$$ (4)

It is also possible to specify a user-defined function for the computation of convective flux. In this case, the user-defined mass flux is assumed to be of the form

$$F=\int_{S}\rho \vec{u}\cdot d\vec{S},$$ (5)

where $d\vec{S}$ is the face vector area.

For NTP-enriched multiphase flow simulation, the CFD solver solves transport equations for two types of scalars: per phase and mixture. For an arbitrary kth scalar in phase-1, denoted by $\phi_{l}^{k}$ , the transport equation inside the volume occupied by phase-1 should be solved

$$\frac{da_{l}\rho_{l}\phi_{l}^{k}}{dt}+\nabla \cdot \left(da_{l}\rho_{l}{\vec{u_{l}}\phi }_{l}^{k}-a_{l}\Gamma_{l}^{k}\nabla \phi_{l}^{k}\right)=S_{l}^{k}\;k=1,\ldots ,N,$$ (6)

where $a_{l},\rho_{l}$ , and $\vec{u_{l}}$ are the volume fraction, physical density, and velocity of phase-1, respectively. $\Gamma_{l}^{k}$ and $S_{l}^{k}$ are the diffusion coefficient and source term, respectively, which must be specified. In this case, the scalar $\phi_{l}^{k}$ is associated only with one phase (phase-1) and is considered an individual field variable (phase-1).

The mass flux for phase-1 is defined as

$$F_{l}=\int_{S}a_{l}\rho_{l}{\vec{u}}_{l}\cdot d\vec{S}.$$ (7)

If the transport variable described by the scalar $\phi_{l}^{k}$ represents a physical field that is shared between phases, or is considered the same for each phase, then it is suggested to treat this scalar as being associated with a mixture of phases (gas, solid, or emulsion in an NTP system). In this case, the generic transport equation for the scalar is

$$\frac{d\rho_{m}\phi^{k}}{dt}+\nabla \cdot \left(\rho_{m}{\vec{u}}_{m}\phi^{k}-\Gamma_{m}^{k}\nabla \phi^{k}\right)=S^{k_{m}}\;k=1,\ldots ,N,$$ (8)

where mixture density $\rho_{m}$ , mixture velocity ${\vec{u}}_{m}$ , and mixture diffusivity for the scalar ${k\;\Gamma }_{m}^{k}$ are calculated as follows:

$$\rho_{m}=\sum_{l}a_{l}\rho_{l},$$ (9)

$$\rho_{m}{\vec{u}}_{m}=\sum_{l}a_{l}\rho_{l}{\vec{u}}_{l},$$ (10)

$$F_{m}=\int_{S}rho_{m}{\vec{u}}_{m}\cdot d\vec{S},$$ (11)

$$\Gamma_{m}^{k}=\sum_{l}a_{l}\Gamma_{l}^{k},$$ (12)

$$S_{m}^{k}=\sum_{l}S_{l}^{k}.$$ (13)

When catalysts are involved in the reactions, it is essential to specify the diffusivity for each material within the individual phases to accurately compute the mixture diffusivity (Matsson, 2022; Stolarski et al., 2018).

**FIGURE 1:**
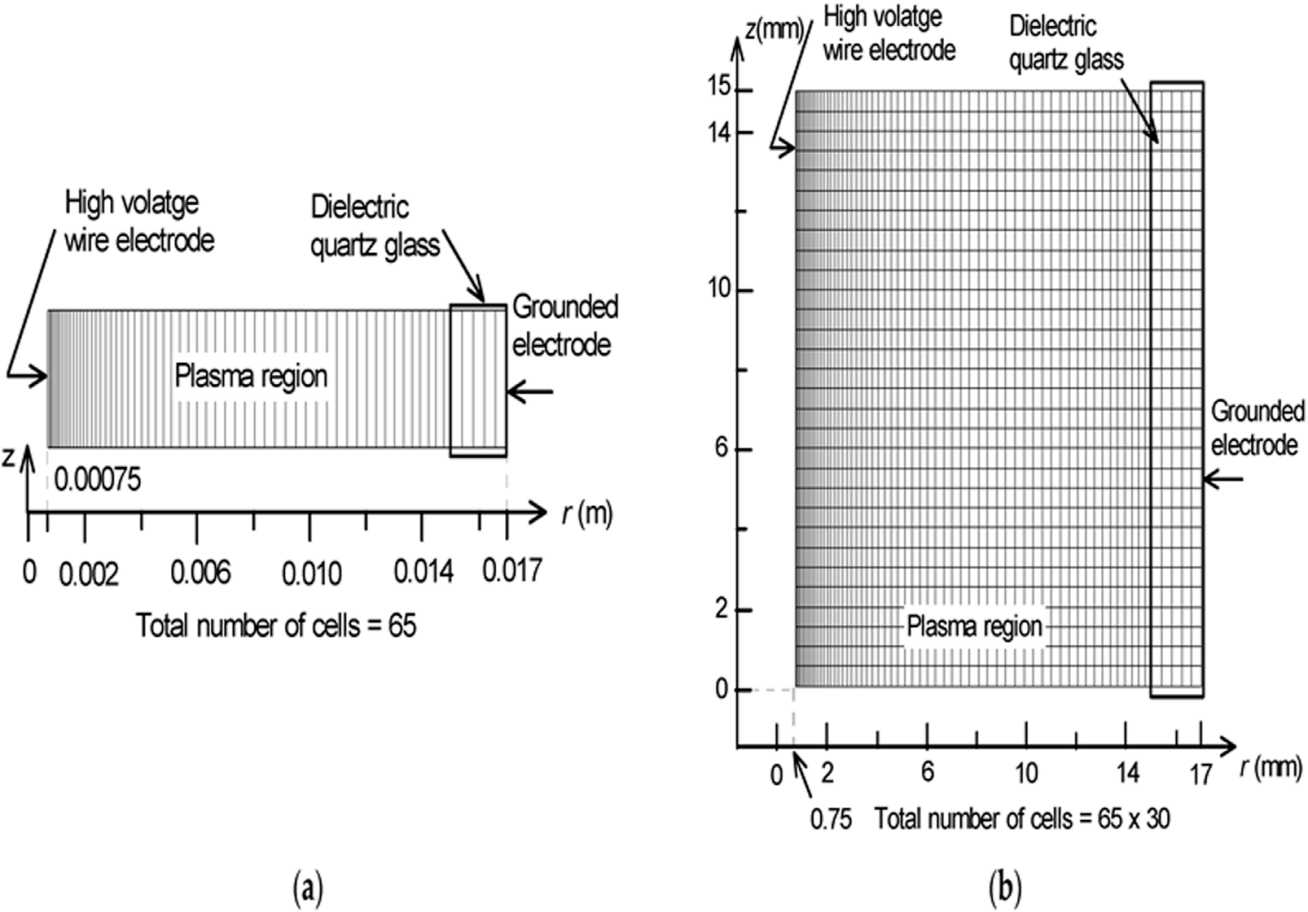
Various computational grid refining methodologies: **(a)** lower cell numbers without tuning for expedited, overarching analysis. **(b)** 2D with higher cell numbers and comprehensive refinement to simulate plasma dynamics within the reactor, incorporating grid refinement impact analysis in areas characterised by pronounced gradients (Okubo, 2023).

Notably, after listing or selecting the sets of necessary equations, successful CFD simulations for plasma-enhanced systems rely heavily on accurate geometry development and mesh generation. Incorrect meshing can result in calculation failures. Computational grid size and cell arrangement are crucial and depend significantly on the system’s shape and type. For instance, non-uniform grid generation may lead to a quasi-one-dimensional model (Figure 1a), where the physical quantities of electrical parameters are averaged across different directions. This particular methodology, although it may not be the most optimal or effective means of thoroughly examining and elucidating the complex and multifaceted architecture that characterizes the discharge phenomena originating from plasma electrodes, nevertheless possesses the capacity to yield valuable and significant insights that can contribute to our understanding of the underlying processes involved. In this case, mesh size is especially important for determining the technical specifications of the NTP system. For example, near the high-voltage wire electrode, where physical gradients are larger, the mesh should be finely refined to allow a detailed analysis of the discharge region and the electric field within the dielectric material.

Hence, the NTP-related energy equations are applicable for illustrating the energy transmission in the fluids, which can be observed in particular grids (Figure 1a). Here, the fluid dynamics and energy transmission models are suggested to be coupled and solved in a time-dependent solver.

A 2D mesh generation for an NPT reactor modelling is shown in Figure 1b. A refined and improved mesh for a cylindrical geometry is generally suggested (Figure 1b) to examine and explain the impact of electrical parameter variations on the reactant fluids.

In a cylindrical synchronised system (Figure 1a), the fluids flowing in multiple directions (r, θ, z) are considered; nonetheless, the slope is measured only in the r direction (∂/∂θ = ∂/∂z = 0). Thus, the model turns into a quasi-one-dimensional model. Consequently, by using this geometry (Figure 1a), the parameter values are defined as averaged values in the θ and z axes. Conversely, if the gradients are defined in both r and z (Figure 1b), where the value stands for ∂/∂θ = 0, then the model becomes asymmetric, and the averaged results trend towards the θ direction. This model is convenient for explaining the comprehensive configuration of nonlinear streamers in the z direction and is applicable for examining the multidirectional reaction dynamics in the NTP environment.

Additionally, heat transfer phenomena in plasma are extremely complex and intricately related to chemical reactions, electromagnetic fields, changes in physical properties, and fluid flow when CFD approaches are implemented for plasma-powered systems (Okubo, 2023). Theoretically, the identified reactor should be modelled as a 3D-geometry to account for the uniformity of the reaction rate in various sections of the system and forms of flow and phase changes of the fluids. However, 3-D simulations are very time-consuming when the kinetics of reactions are integrated for the initialisation of calculations, which is anticipated to require several weeks or even months employing existing software. Because of these computational limitations, a 2D geometry is proposed in almost all studies related to NTP reactor simulations, along with time-dependent analysis.

**FIGURE 2:**
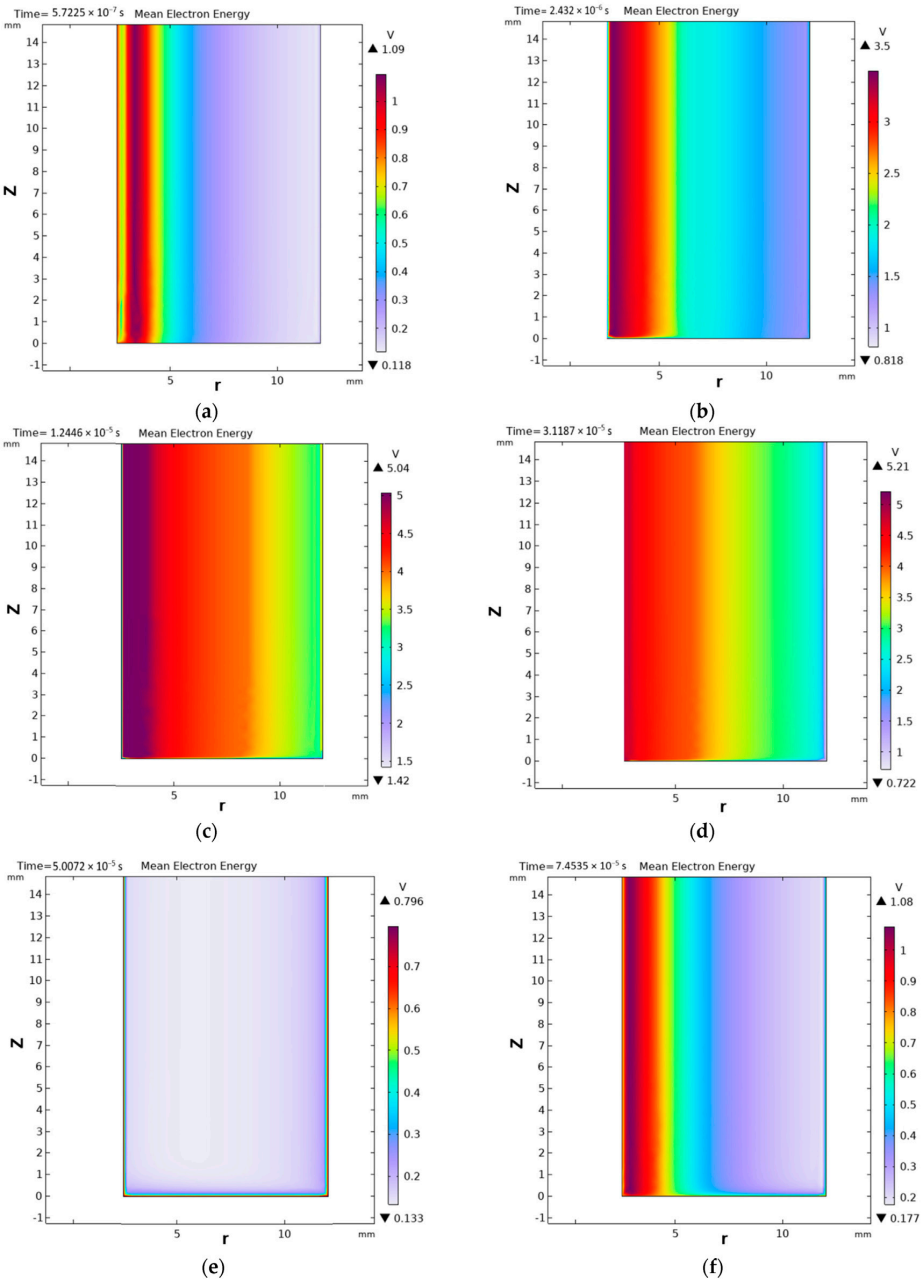
Energy consumption for breaking unsaturated and ionised gas molecules in an NTP reactor (the energy consumption has been shown as the “mean electron energy”) for a CFD model validation across time intervals: a **(a)** 0.57225 μs, **(b)** 2.432 μs, **(c)** 12.446 μs, **(d)** 31.187 μs, **(e)** 50.072 μs, and **(f)** 74.535 μs (Mas-Peiro et al., 2024).

Furthermore, the CFD technique is also very useful to model closed-loop NTP-powered multiphasic reactor simulation by adapting multiple electro-kinetic parameters. For instance, CFD- generated results can provide detailed and clear insights into the impact of NTP reactor design factors, such as dielectric barrier thickness and material, reactor dimensions (length, width, etc.), the inlet fluid flow dynamics, and average electron energy required during high-quality petrochemical production from mixed plastics. Utilising an advanced CFD tool, COMSOL, Mas-Peiro et al. (2024) successfully computed the required energy to break the double bonds of the ionised gases under cold plasma conditions. In their CFD model-validation study, it was discovered that optimum gas atom excitation is achieved at 5.21 V, as illustrated in Figure 2.

Figure 2 depicts the mean electron energy dynamics in a non-thermal plasma reactor over time. These contour plots are essential for analysing plasma evolution and its chemical reaction potential. Initially, the electron energy is localised at the electric field generation terminal. This signifies concentrated plasma initiation and energy input in that area. Over time, electron energy disperses evenly across the reactor’s radius. This indicates that energy is most concentrated near its generation point, tapering off with distance. With increasing iteration periods, energy diminishes consistently along the geometry’s radius (in Figures 2a,b). Electron energy escalates from the initial generation phase until it stabilises. This stabilisation occurs at approximately 12.446 µs, as indicated in Figure 2c. The mean energy peaks at 5.21 V at approximately 31.187 µs (Figure 2d). This peak represents a phase of heightened energetic activity within the plasma. Following this, the mean energy decreases in tandem with the plasma’s overall behaviour. It reaches a minimum at 50.072 µs, marking the end of the first electric potential cycle (Figure 2e). Subsequently, the energy begins to increase again in later time iterations, observed at 74.535 µs (Figure 2f). This cyclical pattern aligns with the applied electric potential. The characterisation of electron temperature mirrors the mean electron energy, enhancing the comprehension of the plasma’s energetic state.

A 2D geometry model was utilised for simulating non-thermal plasma (Figure 2), facilitating fluid flow assessment not achievable with basic 0D/1D models. The model geometry is based on a cylindrical quartz tube reactor serving as the dielectric barrier, featuring an inner copper rod anode and an outer copper mesh cathode. The discharge gap is less than 10 mm. The total DBD length simulated was 160 mm, reflecting the copper mesh length. The model incorporates COMSOL^®^’s Plasma Module and Laminar Flow Module to address governing equations and boundary conditions for fluid and plasma, alongside their multiphysics interactions. Coupled differential equations were resolved for mass, momentum, and energy conservation of various plasma species, including drift-diffusion, heavy species transport, plasma chemistry reaction rates, and Poisson’s equation for the electrostatic field. For fluid modelling, the Navier–Stokes equations were solved to represent the moving fluid’s velocity, pressure, and density. The reactor exhibited laminar flow for all inlet volumetric rates simulated. Plasma was represented as a non-Maxwellian fluid using a two-term Boltzmann equation approximation to derive the electron energy distribution function (EEDF). The integration of CFD in fluid modelling and simulations yielded an insightful comprehension of the interactions between fluid dynamics and plasma phenomena in a DBD reactor. These studies emphasised the significance of factors such as inlet volumetric rate, dielectric material and thickness, and reactor length in enhancing plasma generation and consequently the conversion efficiency of solid polyolefin to gas, notwithstanding the simplifications regarding the role of carrier gases in the simulations.

**FIGURE 3:**
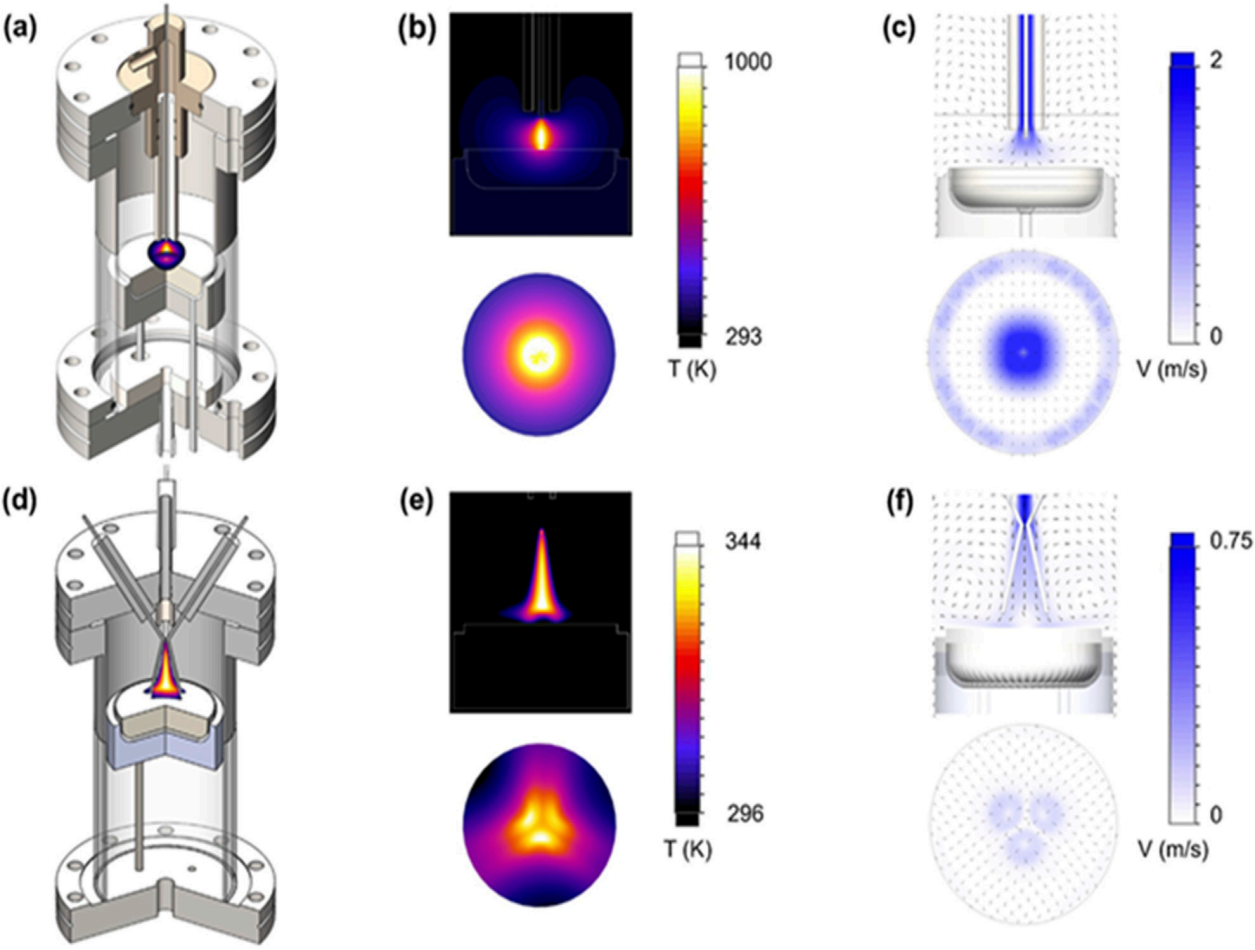
Illustration of computational temperature-dependent fluid reactor models. Transarc reactor components include **(a)** design schematic, **(b)** temperature distribution, and **(c)** velocity distribution for heat dissipation; glidarc reactor components encompass **(d)** design schematic, **(e)** temperature distribution, and **(f)** velocity distribution for heat dissipation (Tabu et al., 2022). Permission for reproduction was granted by Elsevier.

Moreover, CFD-based thermal-fluid models can also predict expected operational characteristics based on design and operating parameters for NTP reactors using complementary discharge technologies such as transarc and glidarc. Factors such as flow rate, electrode-feedstock spacing, and dissipated thermal power (related to voltage) are essential to examine the optimisation of gaseous fuel production from polyolefin. Simulation engineers are recommended to consider the electrode spacing, flow rate, and voltage level as these critical parameters directly influence the reactor performance. The models (Tabu et al., 2022) are designed to predict reactor operations as a function of inflow rate (Q) and the thermal power dissipated by the plasma, correlated with voltage (V). Representative CFD results for thermal-fluid models are shown in Figure 3.

Figure 3 depicts the developed computational geometry of thermal-fluid models, along with velocity and temperature distributions for typical operating conditions, particularly dissipated thermal power (W). In the transarc reactor, temperature distribution (Figure 3b) peaks at the plasma centre and decreases outward radially, reaching ∼750 K on the feedstock surface. The high temperatures in transarc simulations are attributed to the small plasma volume, which leads to greater thermal power per unit volume. The glidarc reactor, on the other hand, shows a three-fold symmetric temperature distribution (Figure 3e), indicating non-uniform heating of the feedstock, with peak temperatures of approximately 300 K. This model projects a larger plasma-feedstock interaction area than the transarc reactor, as depicted in the isosurface temperature distributions in Figures 3a,d.

As the domain of CFD study continues to evolve and progress, it becomes increasingly imperative to engage in a thorough exploration of the potential integration of reaction kinetics modelling algorithms with CFD approaches, with the ultimate goal of significantly enhancing predictive capabilities and optimising the design frameworks of NTP-powered reactors. The concepts surrounding the hybridisation of various models may serve a crucial and transformative function in revolutionising the design processes of multipurpose reaction systems that are specifically aimed at the valorisation of polymeric wastes, thereby ensuring that both efficiency and sustainability are concurrently achieved in a wide array of multi-sectoral applications. The preceding discussion emphasises the paramount importance of ongoing and continuous innovation in the methodologies associated with CFD as this innovation is essential to effectively tackle the numerous challenges that arise in the processing of polyolefin waste while simultaneously working to bolster the sustainability of non-thermal plasma systems.

### 3.2 Reaction chemistry modelling

The key components of plasma chemistry modelling are the species involved and their properties, such as electron impact reactions, transport coefficients, heavy species interactions, and surface reactions. To develop a chemical reaction model for plasma-treated systems in simulations, calculation simplifications are often necessary. Considering all the thermodynamic, chemical, and fluid-dynamic interactions of the inlet and outlet mixtures would be extremely complex. Since gases make up the majority of the compounds in NTP reactors and act as diluents for plasma breakdown, simplifying the plasma characterisation is feasible by defining the properties of the gases. Both pure and mixed gases not only initiate and sustain the plasma but are also the key compounds that influence the plasma in NTP applications. The predominant methodology for converting polyolefins into fuels through the application of NTP involves, initially, the pyrolysis of solid polyolefins, followed by the processing of the pyrolysed product within an NTP environment (Khan et al., 2024b; Khatibi et al., 2025). Therefore, an in-depth understanding of the intricate reaction kinetics and the chemical mechanisms underlying polyolefin pyrolysis is essential; consequently, the formulation of an all-inclusive model represents the most viable approach for scholars engaged in this domain.

**FIGURE 4:**
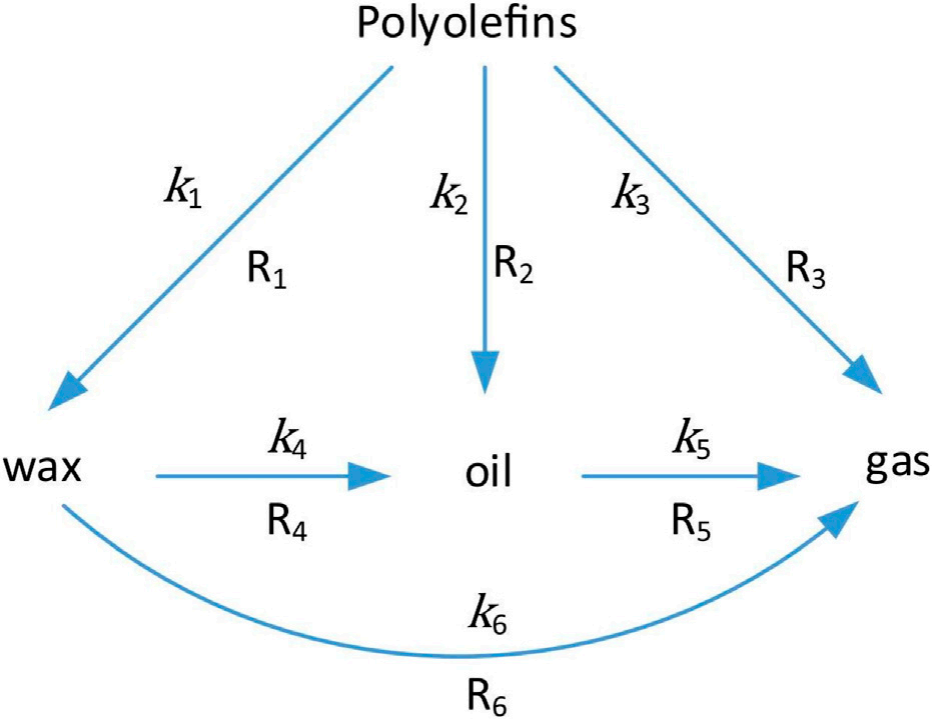
Reaction pathways for the pyrolysis of polyolefins. The designations R1 through R6 denote reaction pathways 1 to 6, while k_1_ through k_6_ signify the associated rate constants. Permission for reproduction was granted by Elsevier (Jiang et al., 2022).

The lumped model has been employed to elucidate the reaction kinetics observed during polyolefin pyrolysis, wherein the changes in the concentrations of both reactants and products are calculated under isothermal conditions, and rate coefficients are deduced from corresponding rate equations. The model necessitates the aggregation of numerous chemical species into a limited number of equivalent “lumps,” which are regarded as homogeneous ensembles, and the implementation of a kinetic model typically employed for individual molecular compounds to represent the lumps. A series of experiments must be performed by varying a range of values of the process parameters while maintaining constant temperatures and leveraging the temperature dependence of the rate coefficient. However, this methodology exhibits a significant drawback regarding the time and resources necessary to conduct a comprehensive set of experiments under isothermal conditions (de Oliveira et al., 2016). Moreover, it necessitates that a sample be heated to a specific temperature, with the reaction subsequently being quenched after varying reaction durations. Another limitation associated with this method is the duration required for heating. For instance, a small tubular reactor utilised by Ramdoss and Tarrer (1998) required 4 min to achieve 450 °C. In the case of a larger reactor utilising electric heating, the time to reach the desired temperature would substantially exceed 4 min, resulting in a considerable extent of reaction having already transpired at temperatures below the target value for the experiment. A model validation study by Jiang et al. (2022) utilised the lumps method under non-isothermal conditions to estimate polyolefin valorisation rate constants. Thermochemically processed polyolefin products were categorised into three “lumps”: wax (W), liquid oil (L), and gas (G). This lumping scheme is applicable to facilitate comparisons of kinetic parameters obtained through the conventional isothermal method. Research study (Jiang et al., 2022) showed that this model-scheme involved a total of six reactions in the overall polyolefin valorisation process, as illustrated in Figure 4.

Throughout this reaction network (Figure 4), polyolefins are thermally cracked into wax, oil, and gas, systematically arranged according to molecular mass. Wax can be cleaved into smaller oil and gas molecules, and oil can also be thermally cleaved to gas. Key assumptions include the following: gaseous products are not subjected to further cracking; all cracking reactions are irreversible; all follow a free-radical mechanism; and the kinetics of the six reactions are characterised by six first-order non-reversible reactions, as delineated by Equations 14–17.

$$\frac{dP}{dt}=-k_{1}P-k_{2}P-k_{3}P,$$ (14)

$$\frac{dW}{dt}=-k_{1}P-k_{4}W-k_{6}W,$$ (15)

$$\frac{dL}{dt}=-k_{2}P-k_{4}W-k_{5}L,$$ (16)

$$\frac{dG}{dt}=-k_{3}P-k_{5}L-k_{6}W.$$ (17)

Here, P, W, L, and G denote the mass fractions of plastics, wax, oil, and gas, respectively, while k_1_ to k_6_ signify the rate constants of the kinetic equations. The developed non-isothermal method transforms Equations 14–17 from time derivatives to temperature derivatives, as specified in Equations 19–22, employing the constant heating rate relationship in Equation 18.

T=βt, (18)

$$\frac{\beta dP}{dT}=-A_{1}e^{-\frac{E_{1}}{RT}}P-A_{2}e^{-\frac{E_{2}}{RT}}P-A_{3}e^{-\frac{E_{3}}{RT}}P,$$ (19)

$$\frac{\beta dW}{dT}=A_{1}e^{-\frac{E_{1}}{RT}}P-A_{4}e^{-\frac{E_{4}}{RT}}W-A_{6}e^{-\frac{E_{6}}{RT}}W,$$ (20)

$$\frac{\beta dL}{dT}=A_{2}e^{-\frac{E_{2}}{RT}}P+A_{4}e^{-\frac{E_{4}}{RT}}W-A_{5}e^{-\frac{E_{5}}{RT}}L,$$ (21)

$$\frac{\beta dG}{dT}=A_{3}e^{-\frac{E_{3}}{RT}}P+A_{6}e^{-\frac{E_{6}}{RT}}W+A_{5}e^{-\frac{E_{3}}{RT}}L.$$ (22)

In this context, A_1_ to A_6_ represent pre-exponential factors, E_1_ to E_6_ denote activation energies for the respective reactions, T is the temperature, and t is the time. Non-isothermal measurements were performed under controlled conditions for polypropylene (PP) and polyethylene (high-density polyethylene (HDPE) and low-density polyethylene (LDPE)), with temperature and lump yields being non-linearly regressed to derive kinetic parameters. The kinetic rate constants obtained revealed patterns that align with those documented in the literature through the isothermal method, although they were lower than values observed under comparable conditions. The computed data, utilising the measured kinetic parameters, aligned with experimental results. The non-isothermal approach developed showcased a significantly quicker technique for determining intrinsic rate constants at increased temperatures. The significance of the mathematical terminology utilised within Equations 14–22 can be observed in alternative contexts (Jiang et al., 2022). The lumping model simplifies reaction kinetics computations but overlooks molecular-scale changes, limiting accurate pyrolysis simulation and generalisation, thus hindering model development for techniques such as NTP to enhance product quality. Consequently, the complex nature of molecules and reactions creates significant challenges in obtaining detailed microscopic insights exclusively through experimental methods (Rahimi and García, 2017).

The advancement of computational technology and sophisticated algorithms is anticipated to facilitate the construction of molecular-level kinetic models, consequently advancing the insight into elaborate reaction mechanisms (Agarwal et al., 2019; Keçeli et al., 2019). The structure-oriented lumping (SOL) methodology serves to represent hydrocarbon molecules and their corresponding reactions through a series of structural units. Within this theoretical framework, reaction networks can be represented using vectors and vector operations, consequently providing a foundational theory for the construction of molecular-level kinetic models (Jaffe et al., 2005). For instance, Ghosh et al. (2006) employed the SOL methodology to forecast the octane rating of gasoline, which is instrumental in comprehending the characteristics of gasoline produced through the application of the NTP technique. It is imperative to acknowledge that the SOL methodology possesses the capacity for extension through the incorporation of additional structural units. Agarwal et al. (2019) devised the bond-electricity matrix (BEM) methodology (Agarwal et al., 2018), which facilitates the simulation of petrochemical processes at the molecular level through a detailed depiction of molecular structures and electron positioning. Furthermore, the molecular-type homologous series (MTHS) and the bond-electron matrix with atomic topology emerged as significant methodologies for molecular-level reaction kinetic models (Gomez-Prado et al., 2008; Joshi et al., 1999). It is significant to note that numerous studies have sought to implement these methodologies in the pyrolysis of polymers, specifically including PP pyrolysis (Harmon et al., 2021; Lele and Ju, 2023). A reaction molecular dynamics-based approach was formulated by Lele and Ju (2023) to examine the influence of mass residence time distribution on product selectivity during the pyrolysis of polyolefins, yielding mass fractions of principal products such as propylene, ethylene, and methane across varying average residence times. Additionally, the application of these models to the pyrolysis of polyolefins, characterised by long chain lengths, presents complexities and computational intensiveness (Harmon et al., 2021).

**FIGURE 5:**
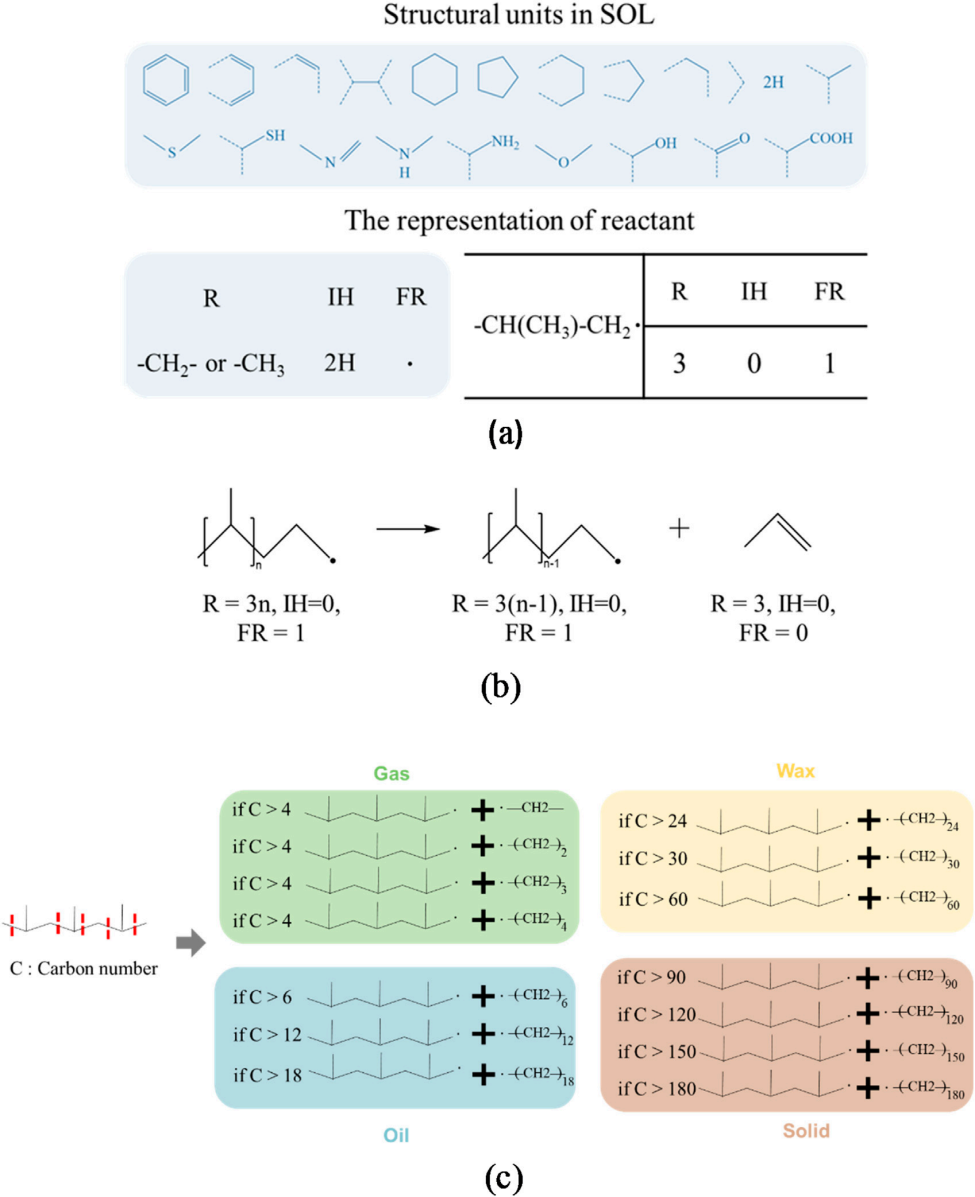
Advanced SOL modeling of molecular dynamics and reaction routes pertinent to the pyrolysis process of polypropylene. **(a)** Depiction of an individual molecule of the polyolefin; **(b)** illustration of the chemical reactions; **(c)** molecular transformation of the products and the reactions involved in the pyrolysis of polypropylene.

Compared with alternative approaches, the SOL methodology provides notable advantages in terms of its straightforwardness and relevance to intricate systems, particularly those featuring polymers with consistent molecular structures. Given the parallels in molecular structures and reaction systems between petroleum oils and polymers, the SOL methodology can be readily adapted by selecting and incorporating structural units, thereby providing a pragmatic framework for the pyrolysis of polyolefin. As stated by Fu et al. (2023), the utilisation of the SOL model involves the creation of structural components that accurately reflect hydrocarbons and free radical intermediates along with their interactions. As shown in Figure 5a, structure vectors link information pertaining to the counts of carbon and hydrogen atoms in separate molecules, thereby generating a molecular matrix representation of all species. Importantly, structure vectors lack the capability to differentiate isomers, primarily indicating products such as gas, oil, and wax by their chain length. In addition, the representation of chemical reactions can be attained through the integration and extraction of structural vectors, as exemplified in Figure 5b, together with reaction coefficients to develop a reaction network. In this case, the reaction model for polyolefin pyrolysis encompasses two components: the selection of reactants and the transformation from reactants to products, which has been established as a critical determinant of the reaction network’s size and simulation accuracy in prior research on polystyrene pyrolysis (Hua et al., 2022). The reaction model is derived from the reaction mechanism, which includes mid- and end-chain carbon–carbon scission and intramolecular hydrogen transfer, to encompass the carbon number distribution of each product (Murata et al., 2002; Savage, 2000). Yet, the reduction of complexity in reaction rules is necessary to reduce the computational demands and time delays associated with intricate reaction networks. Consequently, to preserve significant mechanistic information while constructing a manageable reaction network, so called “14 reaction rules” have been formulated as shown in Figure 5c, with detailed explanations by Fu et al. (2023), primarily addressing chain length alterations and disregarding non-dominant reactions (Ahm et al., 2015). Significantly, the differentiation between primary and secondary reactions is exclusively determined by the selection of reactants.

Additionally, the electrochemistry models may include heavy species reactions such as Penning ionisation and metastable quenching. Surface reactions must also be defined, automatically setting a flux boundary condition for heavy species that corresponds to their velocity, as generally provided in the software database. In this approach, ions that contact the NTP-electrode wall are assumed to revert to neutral gas atoms, transferring their charge to the reactor wall. Secondary emission parameters can also be incorporated for each boundary, with experimental data typically used for secondary emission coefficients, while the mean energy of secondary electrons can be calculated based on the reactant’s ionisation energy.

**FIGURE 6:**
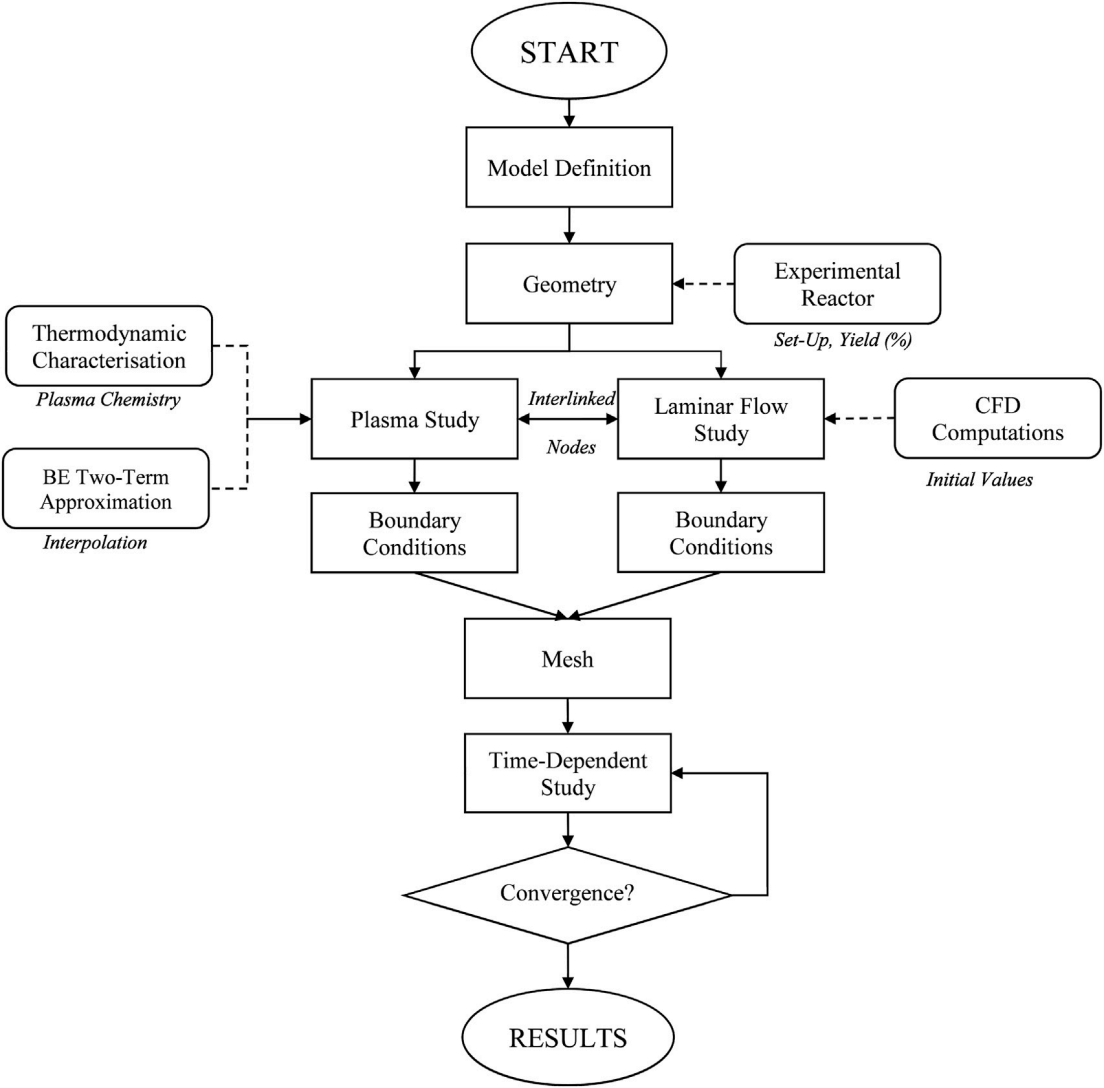
Steps for modelling the system and reactions in a multiphasic stream processed by NTP (Mas-Peiro et al., 2024; Rahimi and García, 2017).

For the simplification of the plasma chemistry model, an accurate molecular model of the gas with thermodynamic characterisation must be defined. This enables a clearer understanding of molecular changes induced by plasma treatment. The general modelling steps are outlined in Figure 6.

Importantly, during NTP processing, the thermodynamic behaviour of ionised gases can be calculated using the soft-SAFT equation (Alkhatib et al., 2020; Llovell and Vega, 2006), a variant of the SAFT (Statistical Associating Fluid Theory) equation of state (EoS) (Chapman et al., 1989). SAFT is a molecular-based EoS derived from statistical mechanics, which is used to describe the thermodynamic properties of complex fluids and predict project phase equilibria. In soft-SAFT, the Lennard–Jones (LJ) intermolecular potential is used to represent both repulsive and attractive interactions (Johnson et al., 1993). This framework enables the modelling of gas mixtures in a simplified form, using a set of molecular parameters (Beuthe and Chang, 1997).

**FIGURE 7:**
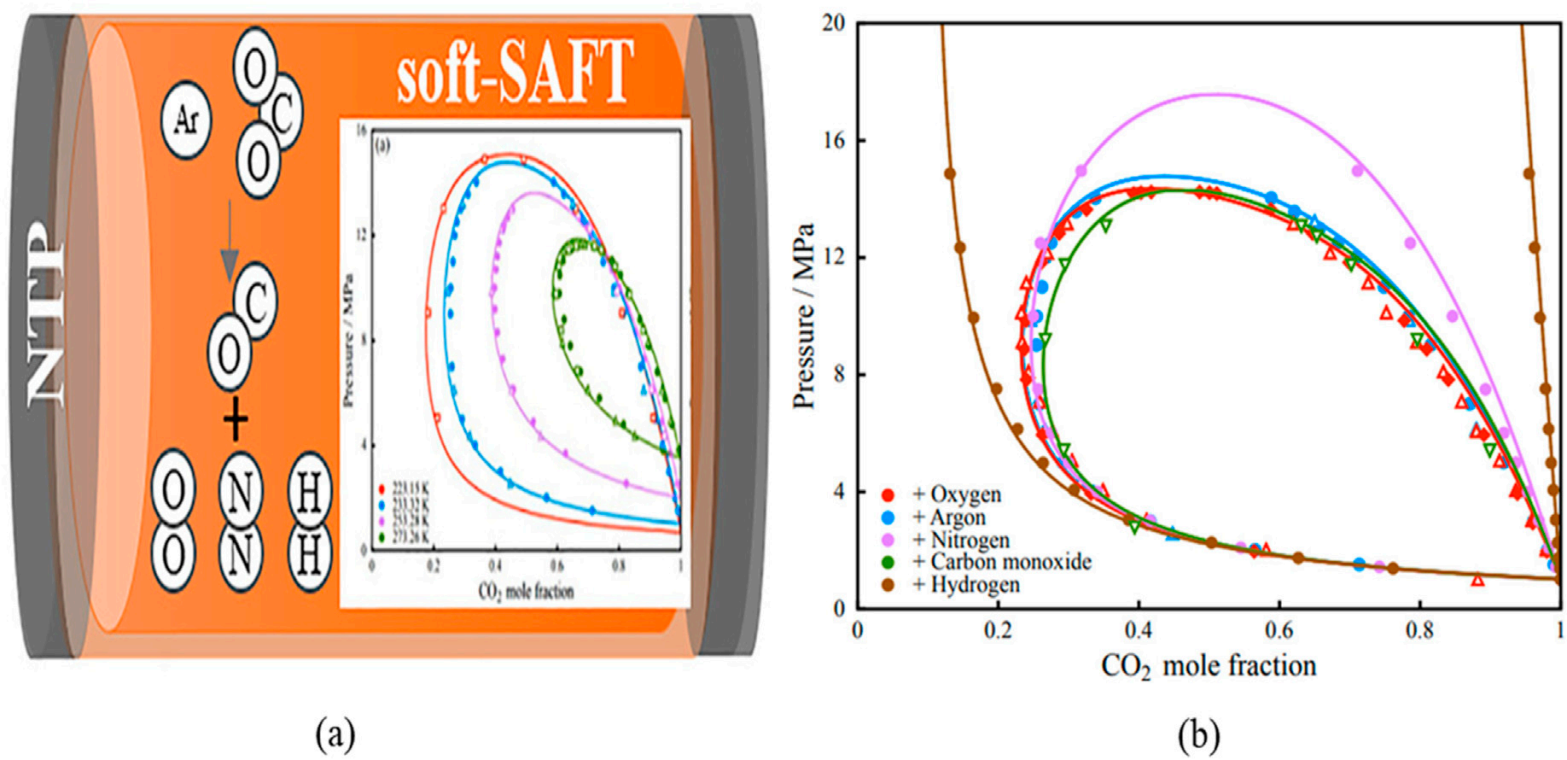
Validation of Soft-SAFT simulation data of the vapor–liquid equilibrium mixed with carbon dioxide during NTP-treated polymers processing in the presence of inert gases; **(a)** argon (experimental data-represented by circles), oxygen (experimental data-depicted by triangles and diamonds), carbon monoxide (experimental data-represented by inverted triangles); **(b)** nitrogen (experimental data-illustrated by circles), and hydrogen (experimental data-denoted by circles). The Soft-SAFT computational results are illustrated by solid lines (Mas-Peiro et al., 2023). Reproduced with permission from ACS.

Key parameters such as homonuclear chain length, monomer–monomer dispersive energy, dipole or quadrupole moments, and segment fractions are critical for the NTP processing of polyolefins. Recently, NTP-treated gas mixtures have been modelled effectively using SAFT, with research findings detailing derivative properties, density behaviour, coefficients, and phase behaviour of binary mixtures in an NTP environment. The results (Mas-Peiro et al., 2023) from SAFT modelling offer a comparative analysis of different gas mixtures, providing insights into their thermodynamic behaviour, as illustrated in Figure 7. The findings show that various gas mixtures behave similarly when CO_2_ concentration is higher in the vapour phase, although notable differences occur in the liquid phase, affecting the phase envelope.

In this case, while the impact of inert gases such as argon (Ar), CO, and O_2_ remains comparable, the introduction of nitrogen (N_2_) significantly increases the system’s vapour pressure. Hydrogen (H_2_) has a significant impact because of the small size of its molecules. These variations can be traced back to the soft-SAFT molecular parameters.

**FIGURE 8:**
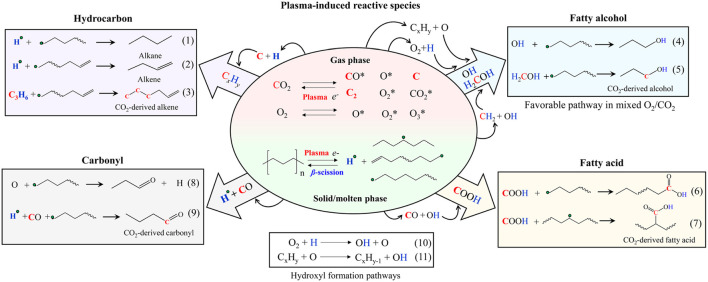
Proposed pathways for the gaseous NTP plasma treatment of solid polyolefins (Radhakrishnan et al., 2024). Used with permission from The Royal Society of Chemistry (RSC).

According to technical surveys, 25 chemical species, 197 phase (mainly gas) reactions, and 21 surface reactions can be considered for study by considering their kinetics in the NTP environments (Bogdanov et al., 2003; Ralchenko, 2005). For example, the reactions during NTP processing of PE with the presence of reactive gas (CO_2_) can be observed in Figure 8. Here, due to NTP discharge, the excited electrons in the reactor could break the C–H and C–C bonds of PE, which results in the formation of hydrogen radicals and shorter-chain hydrocarbons. Later on, β-scission causes the radicalisation of the hydrocarbon and generates alkenes and hydrogen radicals. On the other hand, radical hydrocarbons can be saturated and may produce alkanes (Equation 1) and alkenes (Equation 2) in Figure 8.

Consequently, the C_x_H_y_ radicalisation occurs primarily due to the influence of C (originated from gas, CO_2_) and H (generated from PF). The secondary radicalisation takes place because of the auxiliary coupling reaction with the radicals from hydrocarbons (Equation 3; Figure 8). The fatty alcohol originates due to reactions between PE-originated H and CO_2_-originated O, which form OH and thus promote conjoined hydrocarbon radicals (Equation 4; Figure 8). Likewise, C atoms and OH groups can produce alcohol derivatives can also form through H_2_COH (Equation 5; Figure 8). Fatty acids form as a result of the reaction of CO and OH with hydrocarbon radicals (Equations 6, 7; Figure 8). Carbonyl-based products (Equation 8; Figure 8) and hydrocarbon radicals (Equation 9; Figure 8) may be generated. Continuous plasma discharge can produce excess O and H in CO_2_/O_2_ (Equations 10, 11; Figure 8), will extensively develop OH formation, stimulating the alcohol-forming reactions, and will decrease hydrocarbons. It is worth mentioning that the combination of reactive gas with the NTP technique can synergistically improve plastic conversion (Radhakrishnan et al., 2024). The theoretical model that was developed meticulously integrated the intricate chemistry associated with four distinct electrochemical vectors, specifically encompassing electrons, the pre-treated state of inert gases, the effective excited states of reactive gases, and various gas ions. Additionally, the model comprehensively accounted for a range of electron impact reactions, which included elastic scattering, excitation processes, ionisation phases, and detailed interactions among heavy species, which featured prominent phenomena such as Penning ionisation and metastable quenching processes. Furthermore, the intricate dynamics of surface reactions, along with the essential parameters governing secondary electron emission, were explicitly defined and incorporated into the overall framework of the model.

Ultimately, the modelling of plasma chemistry within NTP systems exemplifies a complex yet critical effort that necessitates an extensive understanding of the various species present, their interactions, and the thermodynamic characteristics of phase-change reactions (solid–liquid–gaseous mixtures). The reduction of complexity in these models, particularly via the application of computational chemistry frameworks, such as conventional kinetic, lump, SOL and soft-SAFT, facilitates the investigation of intricate chemical phenomena, including ionisation, radical generation, and surface interactions. By meticulously characterising the molecular dynamics of gases and their interrelations during plasma treatment, researchers can acquire significant insights into the kinetics of chemical reactions and the consequent transformations of polyolefin-derived waste (plastic) materials. The exchange of ideas presented here underscores the critical influence of various gas mixtures and their respective concentrations on the thermodynamic behaviour and reaction pathways, ultimately contributing to enhanced methodologies for plastic conversion and other applications pertinent to plasma chemistry.

### 3.3 Electrical parameter model

A thorough investigation into the effect of electrical parameters alongside a careful design of plasma-powered reactors may substantially amplify the efficiency of NTP-based polyolefin process scale-up (Khan et al., 2024b). The NTP-powered reactors can be configured in various designs tailored to specific applications. The utilisation of multiphysics simulation would enable the comprehensible computation by considering the dimensions and geometries of the electrodes, the selection of dielectric materials, and the voltages and frequencies applied. For model validation, the selection of suitable data collection instruments is of utmost importance; for example, electrical probes represent the most precise tools for assessing plasma power and gaining a comprehensive insight into the electrical characteristics of the discharge (Khan et al., 2024a).

Various electrical models have been devised to explain the dynamics of electrical parameters and their influence on the efficiency of plasma systems. These models covered parameters such as the selection of a time-variant resistor, the integration of a comprehensive diode bridge, or the incorporation of a self-commutated converter (SCC) in conjunction with a time-dependent coefficient (Khacef et al., 2006). Alternatively, researchers have formulated mathematical models regarding electrical circuits associated with DBDs, wherein they modified capacitors to investigate the capacitive characteristics of the dielectric barrier and the ionisation of the gas. In these models, the gas was characterised as non-conductive, provided that its voltage remained below a specified threshold value. The parameters within these models demonstrated significant concordance with experimental validations, which were ascertained through the analysis of experimental waveforms corresponding to the voltage and current flowing through the DBD configurations (Pipa and Brandenburg, 2019; Rueda et al., 2019; Florez et al., 2017).

The utilisation of a DBD has been generally suggested for producing NTP conditions within atmospheric environments, which is advantageous for the valorisation of polyolefins (Khan et al., 2024a). DBD discharge often occurs via a streamer breakdown mechanism in a non-uniform electric field. Streamers arise from electron quantities produced by a strong electric field. NTP generation occurs upon achieving the breakdown field, yet electron attachment to heavier particles and reaction product recombination reduce NTP conductivity, leading to its cessation. A decline in electric field strength below the breakdown threshold negatively impacts discharge and NTP production. An electrical model may be employed to ascertain the various power factors distributed by plasma generation systems through the utilisation of an automated control system. In this particular instance, the model presumes that the plasma in the discharge gap (electrode-feedstock spacing), along with the feedstock and dielectric, can be represented as parallel-plate capacitors connected in series. On the basis of this assumption, the electrical capacitances of each element in the plasma circuit are defined by their geometric configurations as follows:

$$C_{f}=\frac{A_{f}}{h_{f}}\kappa_{f}\varepsilon_{0},$$ (23)

$$C_{d}=\frac{A_{d}}{h_{d}}\kappa_{d}\varepsilon_{0},$$ (24)

where *C*_*f*_ and *C*_*d*_ are the capacitances of the feedstock and dielectric, respectively; *ε*_0_ is the permittivity of free space; and *κ*_*f*_ and *κ*_*d*_ are the dielectric constants of the feedstock and dielectric, respectively. It is worth mentioning that the ‘dielectric constant’ value should be defined based on the type of material used for NTP processing; for example, for LDPE, the value is 2.2–2.35 MHz (Khouaja et al., 2021).

**FIGURE 9:**
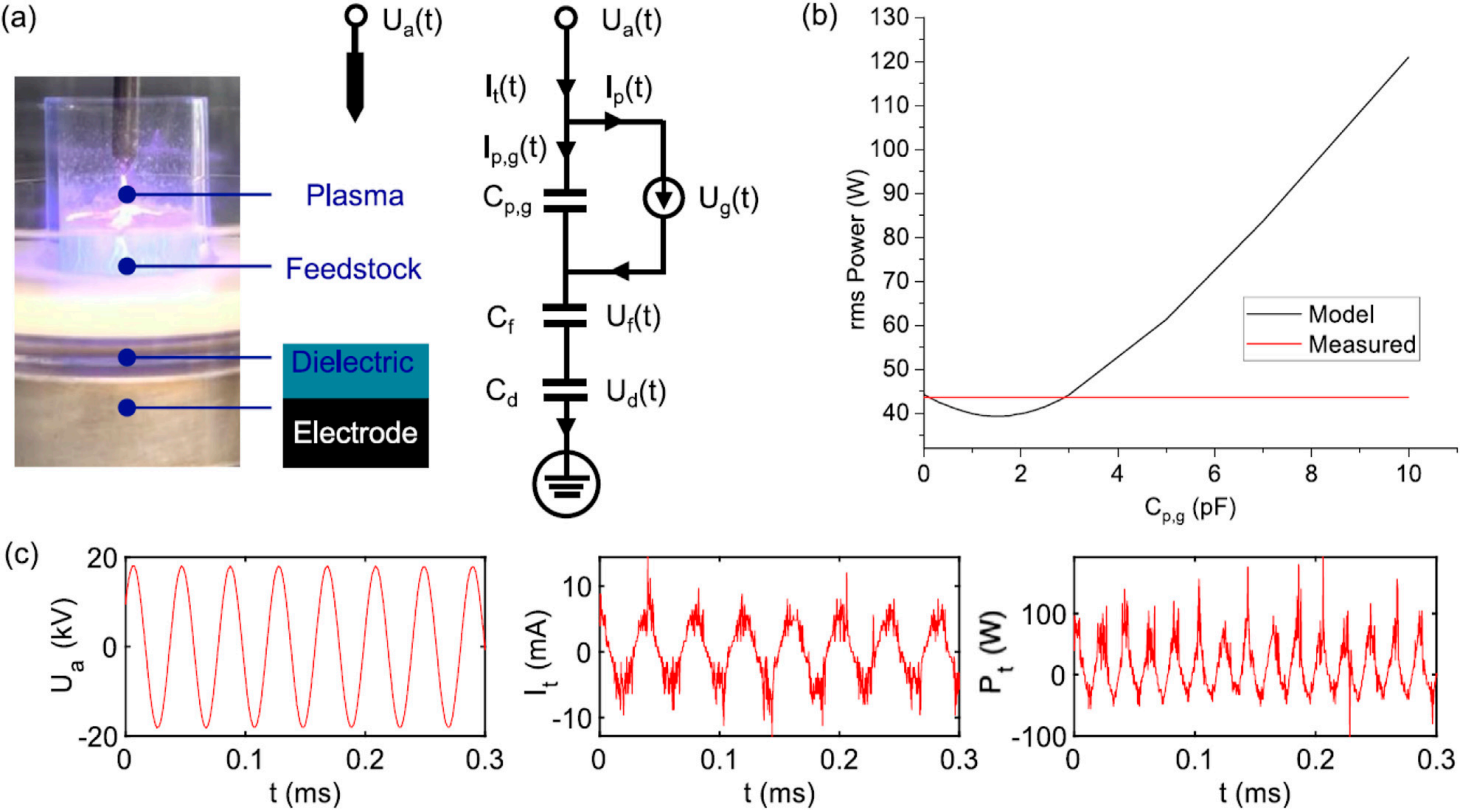
DBD electrical model. **(a)** The operational dynamics of the reactor during the treatment of polymers, accompanied by an equivalent circuit diagram, wherein the feedstock and principal components of the reactor are illustrated as parallel plate capacitors. **(b)** The input power measured in conjunction with the model is depicted as a function of the capacitance of the plasma-gap capacitance Cp,g. **(c)** Oscillograms illustrating the electrical characteristics of input voltage, current, and power (Agarwal et al., 2018). Reproduced with permission from Elsevier.

The electrical model has proven effective for estimating plasma power consumption, and its accuracy can be experimentally validated by analysing the system’s electrical response under a fixed input power. By configuring the dielectric parallel-plate capacitors in a series arrangement and postulating the emergence of a plasma field within the discharge gap (the region situated between the electrode and the feedstock), Tabu et al. (2024) proposed an electrical model focusing on the synthesis of fuel from polyolefins. The approach is illustrated in Figure 9.

In Figure 9a, the structural and numerical modelling of an NTP system for LDPE processing is presented. Figure 9b shows that as plasma power decreases to a minimum, it then increases steadily with an increase in plasma-gap capacitance. This pattern occurs due to variations in the gap displacement current and total displacement current, which influence plasma current and subsequently the plasma power. When the plasma-gap capacitance is zero, the gap displacement current is also zero, so plasma power equals the input power. As the plasma-gap capacitance increases, the gap displacement current increases faster than the total displacement current, leading to a decrease in plasma discharge current and a corresponding reduction in instantaneous plasma power. Eventually, as plasma-gap capacitance continues to increase, the total displacement current surpasses the gap displacement current, resulting in an increase in instantaneous plasma power. The minimum point marks the location where both currents increase at the same rate.

Important equations for developing electrical parameters modelling have been provided below. Using Kirchhoff’s law, the total input voltage *U*_*a*_ *(t)* expressed in terms of plasma voltage *U*_*p*_ *(t)*, voltage across the dielectric *U*_*d*_ *(t)*, feedstock voltage *U*_*f*_ *(t)*, and effective dielectric voltage *U*_*D*_ *(t)* is given by

$$U_{a}=U_{p}\left(t\right)+U_{f}\left(t\right)+U_{d}\left(t\right)=U_{p}\left(t\right)+U_{D}\left(t\right).$$ (25)

The total input current *I*_*t*_ *(t)* is the sum of plasma current *I*_*p*_ *(t)* and the displacement current through the gap *I*_*p,g*_ *(t)*, i.e.,

$$I_{t}\left(t\right)=I_{p}\left(t\right)+I_{p,g}\left(t\right).$$ (26)

The effective capacitance of the feedstock and dielectric *C*_*D*_, given that these are assumed to operate in series, is given by

$$\frac{1}{C_{D}}=\frac{1}{C_{f}}+\frac{1}{C_{d}},$$ (27)

where *C*_*f*_ and *C*_*d*_ are the capacitances of the feedstock and dielectric, respectively. Agarwal et al. (2018) derived the voltage across the feedstock and dielectric, which is given as follows:

$$U_{D}\left(t\right)=\frac{1}{C_{D}}\int_{0}^{t}I_{t,a}\left(t^{\prime }\right)dt^{\prime }+U_{D}\left(0\right),$$ (28)

where *U*_*D*_ *(*0*)* is the memory voltage, which depends on an arbitrarily zero set time (*t* = 0) and is attributed to the memory charges deposited during the preceding AC voltage cycle.

Considering that the negative voltage peak occurs at time zero, *U*_*D*_ *(*0*)* becomes a constant and is defined in terms of the period *T*, i.e.,

$$U_{D}\left(0\right)=-\frac{1}{2C_{D}}\int_{0}^{\frac{T}{2}}I_{t,a}\left(t^{\prime }\right)\;dt^{\prime }.$$ (29)

The plasma discharge current *I*_*p*_(*t*) can be determined from the input current as follows:

$$I_{p}\left(t\right)=\left(1+\frac{C_{p,g}}{C_{D}}\right)I_{t,a}\left(t\right)-C_{p,g}\frac{dU_{a}\left(t\right)}{dt},$$ (30)

where the first and second terms on the right-hand side represent the total displacement current *I*_*v,g*_(t*)* and the gap displacement current *I*_*p,g*_(t*)*, respectively. The total displacement current, sometimes referred to as the external discharge current, is attributed to the effective capacitance of the plasma-gap, feedstock, and dielectric. Hence, it is generally erroneous to assume that the input current is the same as the plasma current, even when the gap displacement current is small and can be neglected.

$$\ln\left(\frac{I_{ij}\lambda_{ij}}{g_{i}A_{ij}}\right)=-\frac{E_{i}}{k_{B}T_{exc}}+D,$$ (31)

where *g*_*i*_ represents the statistical weight of the upper level *i* of the transition considered, *A*_*ij*_ is the transition probability of the emitted spectra, *I*_*ij*_ is the relative intensity of the spectral emission from the upper to the lower states, *λ*_*ij*_ is the wavelength of the emitted spectra, *E*_*i*_ is the excitation energy, *k*_*B*_ is the Boltzmann constant, *T*_*exc*_ is the excitation temperature in electron volt (eV), and D is the data-fitting constant.

The electron number density (*ne*) of the DBD plasma is determined by utilising the method described by Kais et al. (2018), which presents a formula for *ne* that involves the sheath potential Vsh, the gas’s ionisation energy E_ion_ (15.7 eV for Ar), and the electron temperature.

$$n_{e}=\frac{P_{t,rms}}{A_{s}}{\left({\left(\frac{k_{B}T_{e}}{2\pi m_{e}}\right)}^{1/2}\exp\left(\frac{e_{ch}V_{sh}}{k_{B}T_{e}}\right)\left(2k_{B}T_{e}+E_{ion}\right)+\frac{3}{10}k_{B}T_{e}{\left(\frac{k_{B}T_{e}}{m_{i}}\right)}^{1/2}\left(\frac{k_{B}T_{e}}{2}\right)\left|\ln\left(\frac{2\pi m_{e}}{m_{i}}\right)+1\right|\right)}^{-1},$$ (32)

where *A*_*s*_ is the substrate cross-sectional area, *e*_*ch*_ is the elementary charge, *m*_*e*_ is the election mass, and *m*_*i*_ is the ion mass. Consistent with derivation leading to Equation 13, the sheath potential *V*_*sh*_ can be determined using the following expression (Liu and Neiger, 2003; Bashir et al., 2014):

$$V_{sh}=\left(\frac{k_{B}T_{e}}{2e}\right)\ln\left(\frac{m_{i}}{2\pi m_{e}}\right).$$ (33)

From a conceptual standpoint, the model of electrical parameters outlined herein plays an indispensable role in deciphering the dynamics of plasma generation systems, particularly regarding the NTP processing of substances such as LDPE. By representing the plasma, feedstock, and dielectric as parallel-plate capacitors arranged in series, the model proficiently encapsulates the complex interrelations among capacitance, displacement currents, and plasma power consumption. The formulation of pivotal equations, encompassing those for total input voltage, current, and effective capacitance, establishes a comprehensive framework for scrutinising the electrical behaviour of the system under diverse conditions. Moreover, the validation of the model through empirical analysis accentuates its dependability and relevance in the optimisation of plasma processing parameters. The revelations derived from this model not only augment the comprehension of plasma behaviour but also facilitate advancements in the design and regulation of plasma generation systems, ultimately contributing to more efficient and effective processing methodologies across various industrial applications.

**FIGURE 10:**
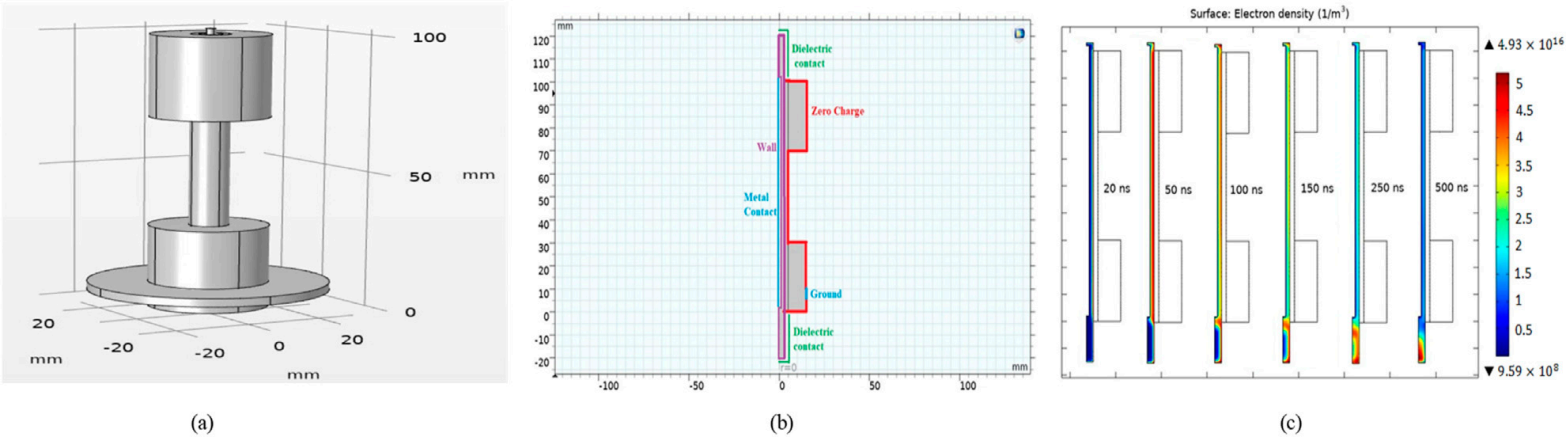
**(a)** Configuration utilised for simulation. **(b)** Discretised mesh incorporating specified boundary conditions. **(c)** Propagation sequences of JDBD emanating from the tube (Roshan et al., 2023).

The integration of multiple devices within plasma powering systems is advantageous for the optimisation of NTP performance. The integration of jet-plasma with DBD has been investigated to assess the influence of insulation selection and integration on the discharge power and product yield. Furthermore, variations in the electric field and frequency were found to be significant. Roshan et al. (2023) proposed a combined jet-dielectric barrier discharge (JDBD) configuration, which constitutes a simulation-driven model designed to design a stable non-thermal plasma system. Their study followed the prescribed protocols of thorough reactor simulation coupled with reaction mechanism modelling, starting with the creation of geometry, progressing to meshing, and finishing with the execution of simulations (Khan et al., 2014). The JDBD geometry (Figure 10a) was meticulously optimised to augment the electric field and analysed charge distribution, thereby facilitating a diffuse and stable discharge in ambient atmospheric conditions. A fundamental aspect of this optimisation process was the “design of effective insulation.” The requisite boundary conditions were delineated by creating a refined mesh, as depicted in Figure 10b. Highly complex conditions, such as electron emission and ionisation, were also scrutinised and explained. The secondary electron emission from the walls imposes definitive boundary conditions, as illustrated in Figure 10b, in the model, while ions are neutralised to the walls through surface reactions. The simulation-derived JDBD architecture, with design parameters including a brass rod of 100 mm length and 2.5 mm diameter, a glass insulator measuring 100 mm in length and 10 mm in diameter, Teflon of 30 mm length and 10 mm thickness, and a brass plate of 3 mm length and 15 mm thickness, is presented in Figure 10a.

The modelling of integrated NTP systems necessitates solving designated equations in addition to the Navier–Stokes equation, which regulates the spatial distribution of charge density. The computation of charge density can be achieved through the resolution of the drift-diffusion equation:

$$\frac{{\partial n}_{e}}{\partial t}+\nabla \cdot \Gamma_{e}=S_{e},$$ (34)

$$\Gamma_{e}=-\left(\mu_{e}{En}_{e}+D_{e}\nabla n_{e}\right),$$ (35)

where Γ_e_ denotes the flux density, S_e_ represents the net source term linked to discharge reactions, E signifies the electric field derived from Poisson’s equation, and D_e_ is the electron diffusion coefficient defined by electron temperature T_c_ and mobility μe as D_e_ = T_c_ · μ_e_.

The mass fraction, w_k_, of non-electron species, comprising ions and neutrals, was obtained as follows:

$$q\frac{\partial }{\partial t}\left(w_{k}\right)+q\left(u\cdot \nabla \right)w_{k}=\nabla \cdot j_{k}+R_{k}.$$ (36)

The diffusive flux vector and species rate expression are denoted as *k* and R_k_, respectively.

The space charge density is determined by plasma chemistry, involving charge Z_k_ and number density n_k_.

$$\rho =q[\sum_{k=1}^{N}Z_{k}n_{k}-n_{e}.$$ (37)

The combination of jet and DBD enhances the electric field and charge distribution for diffuse discharge in atmospheric air without carrier gas flow. The recombination process produces high-density metastable atoms and efficient ionisation when paired with seed electrons (Zhang et al., 2016). The radial electric field component influences electron drift velocity and discharge propagation into the surrounding air. The enhancement of electron density in this zone results in a homogeneous radial discharge. The electrostatic equations were addressed by modelling plasma flow as laminar, with initial settings of velocity and pressure established at 1 m/s and 1 atm, respectively. The dielectric constants for the insulator (Teflon) and glass were set at values of 2.1 and 4.7, respectively. The model was defined with a seed electron density of 10^10^ m^−3^ and an initial mean electron energy of 4 V, with the electric potential set at 0 V. The solutions converged with minimum and maximum element sizes of 2 × 10^−3^ mm and 0.1 mm, respectively, using a time step of 0.1 ns over the interval of 0–400 ns (Figure 10c).

A recent investigation introduced an electrical modelling approach utilising the recursive least squares algorithm (RLSA) to assess and enhance the energy efficiency of modified plasma jets, thereby establishing a comprehensive model for optimising their operational parameters. The plasma-jet combined DBD circuit was represented in accordance with Kirchhoff’s first law, incorporating total current, dielectric current, plasma jet current, capacitance, and resistance to quantify the energy supplied to the reactor, alongside the energy expended during the discharge process. The power delivered was computed by multiplying the discharge voltage by the calculated circuit current. The discharge power, indicative of the energy utilised for ionisation, was ascertained through the resistance inherent in the electrical model. The energy efficiency of the plasma jet was determined by calculating the ratio of the discharge power to the power supplied to the reactor. The reliability of the parameter estimation technique was substantiated by the strong correlation between experimentally measured currents and those derived from the electrical circuit, as well as the notable characteristics of the estimated parameters. A noteworthy discovery indicated that the energy efficiency of the plasma jet could be increased from 75% to 90% through an increase in the applied voltage from 6 kV to 8 kV. The findings offer valuable insights into discharge behaviour and suggest that the proposed model can facilitate the selection of optimal operational conditions to achieve peak efficiency (Hani et al., 2024).

The electrical parameter model is crucial for understanding plasma generation dynamics, especially in non-thermal plasma processing of polyolefin materials, and establishes a platform for additional investigations within this discipline. The imperative to solve the nonlinear equations necessitates the development of a comprehensive computational framework crucial for the detailed analysis of the system’s electrical characteristics across diverse operational scenarios, thus multiplying the fundamental comprehension of the process. The validation of the electrical models through experimental results serves to substantiate their reliability and significance in the optimisation of the electrical parameters associated with plasma processing, thereby reinforcing their practical applicability. Results derived from sophisticated models and experimental validation at laboratory or pilot-scales have demonstrated significant advantages in understanding plasma dynamics and refining the design and parameters of plasma generation systems, in accordance with progressions in computational technologies and scalability of plasma reactors across various applications, including waste valorisation.

### 3.4 Energy and exergy modelling

As previously discussed, NTP is a non-equilibrium plasma that primarily excites electrons and the vibrational modes of heavy particles while largely ignoring the translational and rotational modes. Therefore, NTP sources increase the concentration of reactive radical species instead of increasing the overall temperature (*T*). This reduced specific power consumption in NTP systems enhances the energy efficacy of large-scale waste-to-energy conversion. Additionally, electrode erosion is minimal. Despite NTP’s potential for reforming and valorising polymeric materials, its integration into large-scale waste-to-energy plants remains scarcely explored. Among a limited number of studies, Kwon and Im (2022) formulated a thermodynamic model for a waste-to-energy facility that incorporates the NTP technology to assess the energy-efficient gasification process, taking into account three critical operational parameters: the moisture content present in solid polyolefin waste, the plasma power consumption per unit mass flow rate of waste (Wplasma-NTP [kWh/tonne-waste]), and the cold gas efficiency (CGENTP) [%].

**FIGURE 11:**
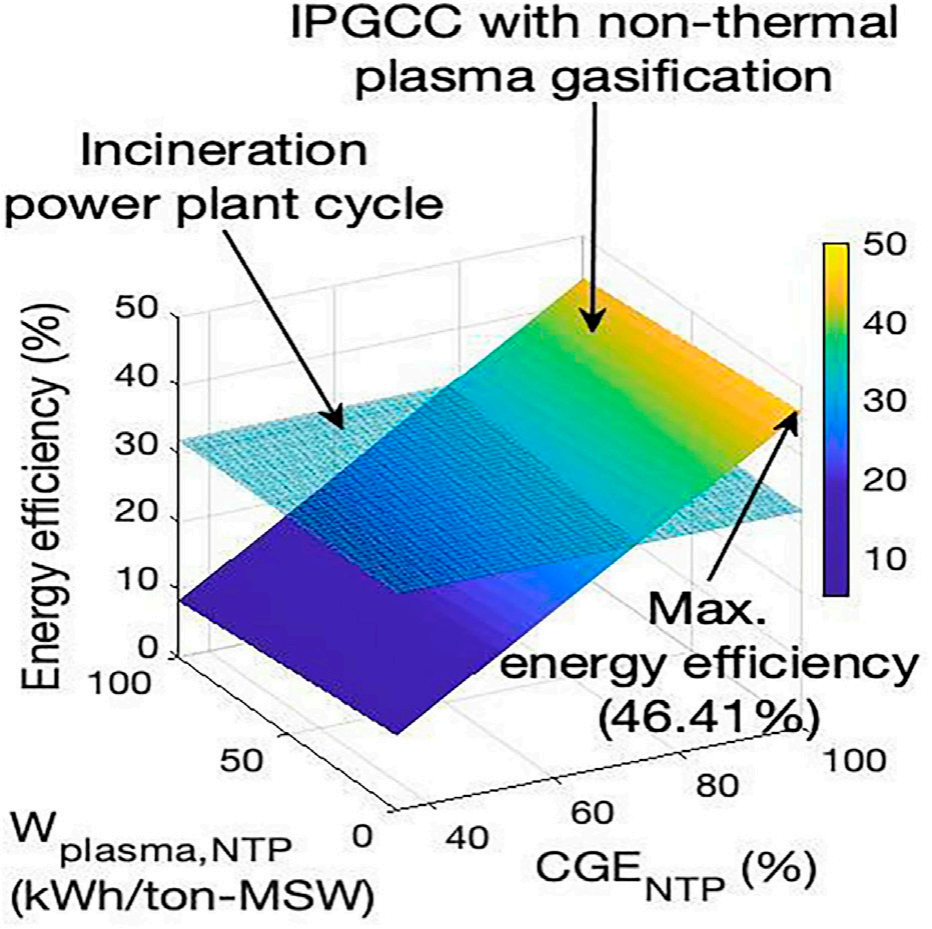
Energy efficiency metrics associated with a non-thermal plasma gasification facility for the processing of mixed plastic solid waste materials (Kwon and Im, 2022). This figure has been reproduced with the authorisation of Elsevier.

Figure 11 illustrates the model’s predictions for an NTP-integrated mixed plastics gasification plant. The model predicts a maximum energy efficiency of 46.41% when CGENTP is at its peak (100%) and Wplasma-NTP is at its lowest (0 kWh/tonne-waste). While these values are idealised, the energy efficiency of a power plant integrated with NTP could reach levels comparable to an incineration power cycle if CGENTP is approximately 75%. This finding is significant and supports the consideration of NTP systems for large-scale power generation from polyolefin waste.

Plasma power consumption is a key factor in determining the overall energy efficacy of a system and can be assessed through sensitivity analysis, particularly using density function-based global sensitivity analysis. This analysis helps evaluate how various input variables affect the energy efficacy of plasma systems. Input variables can be varied randomly; when one input variable, xi, is fixed, while others are randomly altered, the probability distribution of the output variable is given by the conditional density function (fy|xi). To obtain an unconditional density function (fY), input variables should be assumed uniformly distributed across their ranges (fY). The sensitivity indicator, δi, for the input variable (Xi) is derived from the comparison of fY and fy|xi, as shown in Equation 38. δi reflects the average variations in fy|xi relative to fY across the output variable’s range.

$$\delta_{i}=\frac{1}{2}E\left[\int_{Y}\left|f_{y}\left(y\right)-f_{y|X_{i}}\left(y\right)\right|dy\right].$$ (38)

Exergy calculations are crucial for managing power consumption in NTP. Exergy is highly sensitive to process variable adjustments, particularly in solid plastic processing. The degree of exergy loss reveals the irreversibility within each component of NTP-integrated systems. The exergy model should account for T, exergy flows (Ėx), work rate ( $\dot{W}$ ), heat transfer rate ( $\dot{Q}$ ), and reference temperature for every phase, which can be computed using Equation 39.

$$\dot{E}x_{loss}=\dot{E}x_{in}-\dot{E}x_{out}+\dot{W_{in}}-\dot{W_{out}+\left(1-\frac{T_{0}}{T}\right)\overset{˙}{\underset{in}{Q}}-\dot{\left(1-\frac{T_{0}}{T}\right)Q_{out}}}.$$ (39)

The exergy inflow (E˙x_in_) and outflow (E˙x_out_) based on mass flow (m˙) can be determined in terms of enthalpy (h), standard enthalpy (h_0_), entropy (s), standard entropy (s_0_), and chemical exergy (ex_ch_), as shown in Equation 40.

$$\dot{E}x=\dot{m}\left(h-h_{0}+T_{0}\left(s-s_{0}\right)-ex_{ch}\right).$$ (40)

Notably, the ex_ch_ of mixed plastics can be estimated following the method by Huang et al. (2018), while that of a gas mixture is determined using Equation 41, which incorporates the molar fraction (x_i_), chemical exergy (ex_ch,i_) for each gas species, and the universal gas constant.

$$ex_{ch}=\sum x_{i}\cdot ex_{ch,i}+R\cdot T_{0}\cdot \sum x_{i}\cdot \ln\left(x_{i}\right).$$ (41)

The exergy efficiency of both thermal and NTP-driven systems can be computed by choosing the relevant output variables, while the input variables (cold gas efficiency (CGE) and plasma power consumption) are adjustable within given limits. For computing NTP exergy for various types of polyolefin and their deliveries, Equation 41 can be applied.

However, the specific energy input (SEI) represents a fundamental parameter that quantifies the requisite energy input per unit volume or mass in the context of the decomposing petrochemicals under plasma powered conditions. The computation of SEI is carried out by dividing the plasma power by the gas flow rate, and it is typically expressed in units of joules per cubic centimetre (J cm^-3^) or kilojoules per litre (kJ/L). The magnitude of SEI is pivotal in influencing both the conversion rate and the overall energy efficiency attained within plasma systems. The assessment of SEI can be performed utilising Equation 42:

$$\text{SEI}\left(J\;{cm}^{-3}\right)=\text{SEI}\left(\text{kJ}.\;L^{-1}\right)=\text{SEI }\left(\text{eV }\text{per }\text{molecules}\right)==\frac{Power\left(kW\right)}{Flowrate\left(L.\min^{-1}\right)}\times 60\left(s.\min^{-1}\right).$$ (42)

The conversion process, the efficiency of energy utilisation, and the expenses associated with energy consumption can be quantitatively determined through the application of specific methodologies by applying Equations 43–47 (Machmud and Chang, 2023):

$$\eta \left(\%\right)=\frac{\left[FCs\right]in\left[FCs\right]out}{\left[FCs\right]in}\times 100\%,$$ (43)

$$Energy\;efficiency\left(g/kWh\right)=\frac{Massflowrate\left(g.h^{-1}\;\right)\times \eta }{P\left(kW\right)},$$ (44)

$$Energy\;efficiency\left(L.{kJ}^{-1}\right)=\frac{\eta }{SEI\left({kJ.L}^{-1}\right)},$$ (45)

$$EC\left(\frac{kJ}{mol}conv.\right)=\frac{SEI\left(\frac{kJ}{L}\right)24.5\left(L.{mol}^{-1}\right)}{\eta }.$$ (46)

It is imperative to acknowledge that the numerical value of 24.5 L mol^-1^ is exclusively applicable under the conditions of 298 K and 1 atm.

$$EC\;\left(\frac{eV}{mol}\;conv.\right)=Energy\;cost\left(kJ.{\left(molconv.\right)}^{-11}\right)\times =\frac{Power\left(kW\right)}{Flowrate\left(L.\min^{-1}L\;\right)}\times 60\left(s.\min^{-1}\;\right).$$ (47)

**FIGURE 12:**
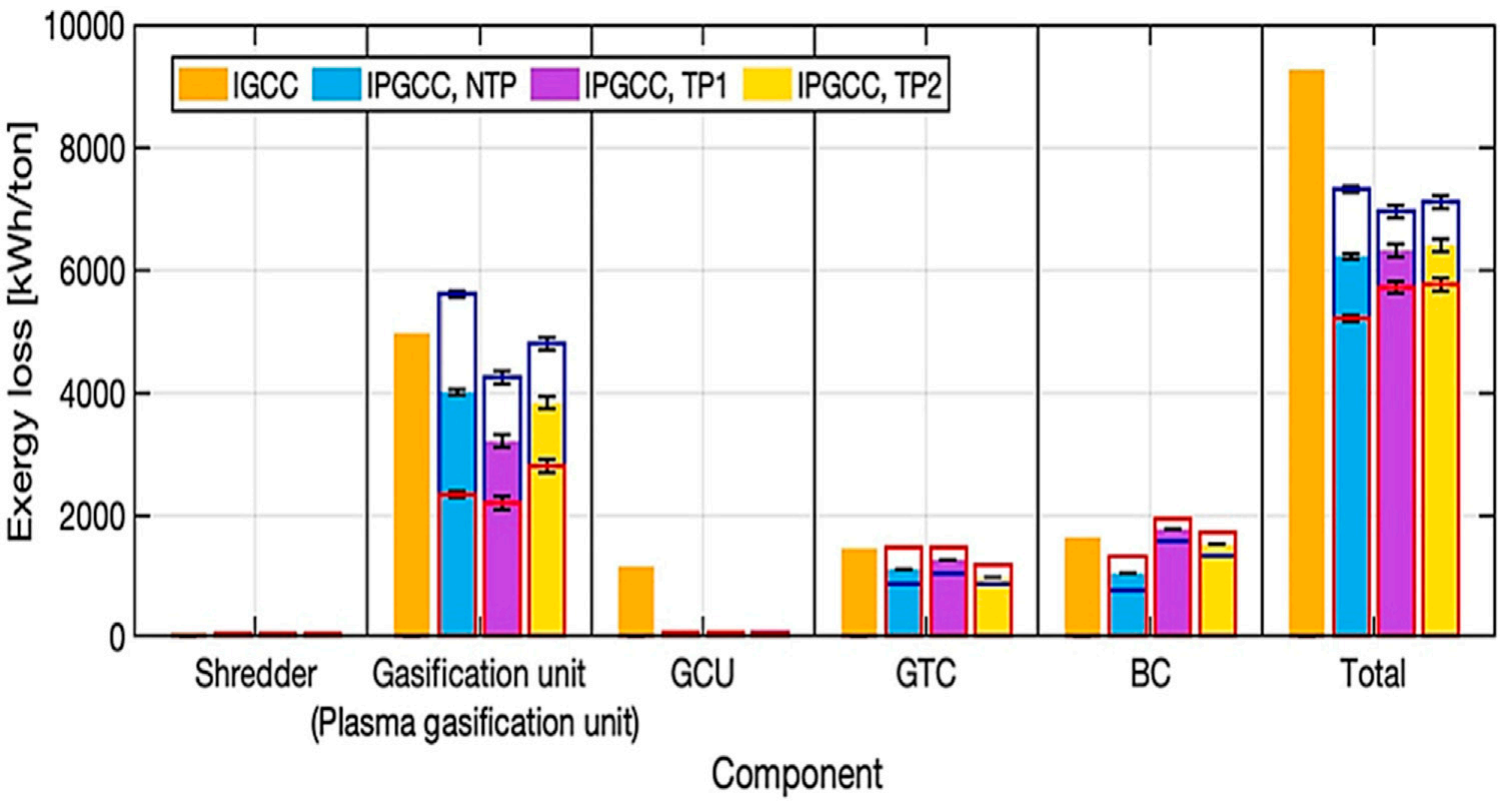
The simulated outcomes of the exergy dissipation for an integrated gasification combined cycle (IGCC) and the integrated plasma gasification combined cycles (IPGCCs) featuring two-tier NTP, one-tier thermal plasma (TP1), and dual-stage thermal plasma (TP2) (Kwon and Im, 2024). Reproduced with consent from Elsevier.

However, research has been conducted to calculate the exergy for NTP processing of waste polyolefins, as shown in Figure 12.

Figure 12 presents the estimated exergy losses for various sections of the IGCC and IPGCC systems. The blue and red bars signify the exergy losses at the lowest and highest CGE values, respectively, while the filled bar indicates losses at a medium CGE value. The black error bars display exergy fluctuations at the extreme values of plasma power consumption. The gasification unit exhibited the highest exergy loss, where char or slag contributed to this loss. A higher CGE reduces exergy loss by increasing the exergy outflow. Additionally, the reduction in exergy loss in the gasification unit was more pronounced with higher CGE than with lower plasma power consumption. As observed in the energy analysis, variations in CGE caused greater fluctuations in exergy loss in IPGCC units than did changes in plasma power consumption in the gasification unit. The exergy loss differences at the extremum CGE for NTP, TP1, and TP2 were 3227.6, 2051.7, and 2011.2 kWh/tonne, respectively, while the differences at extreme plasma power consumption were 100, 200, and 210 kWh/tonne for each system. The fluctuations caused by CGE and plasma power consumption were more than tenfold. This highlights that the NTP-integrated plasma gasification unit can recover significantly more energy at the gasification stage, which can only be accurately predicted through a well-developed model.

The sensitivity analysis concerning the energy feasibility of plasma power consumption, alongside its correlation with CGE, elucidates pivotal insights into the operational parameters that affect energy efficiency within NTP-driven gasification methodologies. Moreover, the utilisation of exergy calculations affords a more profound comprehension of energy dissipation and irreversibilities inherent in the system, thereby underscoring avenues for potential enhancement. As the energy landscape undergoes continuous transformation, an in-depth investigation into NTP systems within extensive waste-to-energy applications is imperative to actualise their comprehensive potential and foster sustainable energy practices. This section establishes a foundational framework for future investigations aimed at optimising the integration of NTP and augmenting the overall efficacy of waste-to-energy conversion technologies.

In addition to the systematic efforts to develop various models by adopting extensive data concerning the applications of the NTP technique across diverse approaches to the processing of waste polyolefins. Some experimental studies have been reported on NTP applications in processing discarded polyolefins under NTP conditions. NTP-based approaches have demonstrated potentially higher energy efficacy and selectivity compared to traditional TP (Fridman et al., 1999; Mohsenian et al., 2019; Snoeckx and Bogaerts, 2017; Wang et al., 2017). Additionally, NTP processes that function at atmospheric pressure are particularly appealing because they offer lower capital and operational costs (e.g. eliminating the need for vacuum systems) and compatibility with other unit processes (Bruggeman et al., 2017). Yao et al. (2021) investigated polyethylene hydrogenolysis in an atmospheric-pressure NTP reactor using a DBD system over solid catalysts, employing H_2_ and Ar as the working gases. They achieved over 95% selectivity for lower alkanes (C1–C3) with less than 5% of unsaturated hydro Cs. Their findings also revealed that adding a catalyst (Pt/C or SAPO-34) improved energy efficiency without significantly affecting the product formation rate. Similarly, Aminu et al. (2020) employed a two-stage pyrolysis/low-temperature plasma catalytic process, also based on DBD, to generate H_2_ and syngas (primarily H_2_ and CO) from plastic waste. They found that low-temperature plasma improved gas production and H_2_ yield compared to a catalyst-only process, with syngas selectivity peaking after 1 min before decreasing because of dominant pyrolysis reactions. Diaz-Silvarrey et al. (2018) used N_2_ DBD to pyrolyse high-density polyethylene, noting an increase in syngas production from 15 wt% to 44 wt% at 600 °C. Xiao et al. (2023) recovered H_2_ and aromatics from polypropylene waste through plasma-catalytic pyrolysis, observing an 18 wt% increase in gas products with 4.19 mmol/g H_2_ produced. Ahmed et al. (2009) reviewed plasma-based hydrocarbon decomposition methods and recommended that NTP could serve as a viable alternative to traditional catalytic processes. Although these studies show promising results, they remain limited to laboratory-scale research, and the potential for scaling up NTP for plastic waste valorisation relies heavily on further development of reaction modelling and reactor simulation (Khan et al., 2024a; Khan et al., 2024b), which remains mostly unexplored.

However, based on the above discussion, it can be summarised that the selection of calculation tools for solving the necessary equations is vital when the specific material characteristics need to be defined. Herein, applied software and the type of polyolefin studied have been provided in Table 2.

**TABLE 2:** Materials, methodologies, parameters, and computational tools utilised for the modelling of the NTP process in relation to polyolefin waste materials.

Type of polyolefin studied	Method of simulation/computational tool	Model parameter calculated	Reference
Biodegradable packaging materials	Finite element method (FEM)/COMSOL Multiphysics	(I) Electric field (ii) Relative permittivity (iii) Initial voltage (iv) Electrode position (iv) Polymer particle shape and size	Chankuson et al. (2023)
Mixed plastic waste	Thermodynamic modelling (Gibbs free energy minimisation (GEM) method)/MATLAB EES (engineering equation solver)	i. Plasma power consumption ii. Energy and exergy analysis	Kwon and Im (2024)
LDPE(low-density polyethylene)	Computational fluid dynamics (CFD) and thermal-fluid models (TEM)/SolidWorks	i. Electrode-distance ii. Flow fluid (gas) iii. Dissipated thermal power iv. Voltage level of plasma	Tabu et al. (2022)
Polyethylene	Wertheim’s first-order thermodynamic perturbation theory (TPT1) for molecular level reaction modelling/Soft-SAFT digital platform	i. Single-phase and phase equilibria of gas mixtures ii. Molecular parameters	Rahimi and García (2017)
LDPE	CFD/SolidWorks	i. Discharge gap (ii) Feedstock (iii) Dielectric value	Agarwal et al. (2018)
Plastic waste mixed with municipal solid waste	Thermodynamic modelling energy/STANJAN, WebBook, Thermoflow’s GT PRO^®^	i. Thermodynamic properties of these gas mixtures in NTP ii. Energy and exergy analysis	Lele and Ju (2023)
Plastics from municipal solid wastes	CFD/ANSYS	i. Equivalence ratio (ER) ii. Steam to fuel ratio (SFR) iii. Input plasma power	Ismail et al. (2019)
Solid plastics waste	Gibbs energy minimisation method/Aspen Plus	(i) Mixture of feedstock (ii) Temperature distribution (iii) Steam flow	Mazzoni and Janajreh (2017)
Solid plastics mixed waste	Species transport/Aspen Plus	(i) Molar fraction gases (ii) Gasification temperature	Sakhraji et al. (2022)
Solid medical waste	CFD and Monte Carlo Radiation model/ANSYS CFX	(i) System temperature (ii) Energy balance	Sharma et al. (2020)
Solid polymeric wastes	Monte Carlo collision/VSim simulation code	(i) Electron density (ii) Dielectric constant (iii) Reactive species in the vicinity	Zhang et al. (2015)
Food package waste	Statistical optimisation/Stat-Ease	Power discharge rate, discharge interval, power frequency, and power intensity	Khan et al. (2024b)

Table 2 clearly conveys explicit and comprehensive information regarding the various types of polyolefin processing methodologies that have thus far been subjected to investigation through the development of sophisticated modelling techniques and advanced simulations. For system or reactor simulation, complex equations in CFD tools are utilised to study multiphase fluid phase transitions due to process parameter changes. Among the software tools that have been utilised for these analytical endeavours are ANSYS, Aspen Plus, and COMSOL Multiphysics, which have proven instrumental in facilitating these analyses. Furthermore, in addition to the aforementioned applications, for exploring reaction kinetics, conducting molecular-level modelling, and optimising various processes, a diverse array of software platforms, including MATLAB, STANJAN, the NIST Chemistry WebBook, Thermoflow’s GT PRO, and State-Ease, have been extensively leveraged and utilised within the academic and industrial research communities.

## 4 Importance of performing a comprehensive literature survey on the modelling and system simulation of the polyolefin process energised by non-thermal plasma

It has become increasingly evident that the modelling and simulation of reaction mechanisms and the NTP system play an extraordinarily significant role due to their boundless potential contributions to both the generation of renewable energy and the reduction of environmental pollution. It is rather disappointing that there has been a rather minimal amount of effort dedicated to conducting comprehensive literature reviews that pertain to the application of non-thermal plasma within the critical intersection of the environmental and energy sectors. As a direct result of this oversight, the intricate details surrounding the dimensions of modelling and simulation have, regrettably, not been accorded the level of consideration that they truly deserve within the broader context of ongoing research and development.

In a mini-review, Abdelaziz et al. (2025) elucidated the advancements in atmospheric-pressure NTP plasmas for NOx production, emphasising yield, energy efficiency, and future applications in power-to-X and renewable energy. To improve plasma-based NO_x_ production, various modelling strategies for optimising reactor design and gas flow are proposed. Key modelling parameters include plasma channel design, effusion nozzle simulations, electrical circuit models, and tangential gas flow. Techniques such as Rayleigh scattering facilitate *in situ* measurements of gas temperature and species densities, providing crucial data on spatial distributions. These diagnostics have uncovered significant insights, including a doughnut-shaped NO distribution, which informs strategies to reduce backward reactions through optimised reactor geometry. CFD and reaction kinetic modelling are essential for deriving insights into plasma NO_x_ production that are challenging to capture experimentally (Abdel et al., 2024).

Nippatlapalli and Ganta (2024) and Zeghioud et al. (2020) performed a review on NTP technology for water treatment, examining mechanisms, reactor designs, active species, and combined processes. Both reviews underscored the increasing interest in this technology due to its efficacy in degrading harmful organic compounds and its potential for low energy use and operational simplicity. Key process parameters, such as input power, pollutant concentration, water conductivity, reactor design, electrode positioning, and feed gas composition, have been identified for optimising NTP-based water treatment. They concluded that the development of reaction modelling and reactor simulation for electrochemical processes will emerge as a promising technology for water treatment, capable of generating various reactor designs and combining conventional water treatment methods, but requiring further research efforts. Nonetheless, challenges concerning non-linear process comprehension and economic feasibility for large-scale applications must be resolved for broader implementation.

Ahmed et al. (2024), Anuntagool et al. (2023), Herian et al. (2021), and Perinban et al. (2019) reviewed the NTP–liquid interaction, NTP-DBD, and NTP-pulse combinations, highlighting their generation and effectiveness in microbial inactivation in food applications. It examined NTP performance factors and their effects on food quality. The authors emphasised the need for further optimisation studies to reduce adverse effects on food quality, including lipid oxidation and changes in phenolic compounds and sensory characteristics during NTP treatment. This requires adjusting processing parameters to ensure effective microbial inactivation while preserving product quality. Although the non-thermal plasma treatment offers significant potential for food decontamination, future research should prioritise optimising treatment parameters, understanding its food quality impacts, and addressing practical issues related to cost, safety, and waste management for broader commercial use.

A literature survey by Birk et al. (2024) revealed the efficacy of NTP for treating silica-based dental ceramics as a safer alternative to hydrofluoric (HF) acid etching. NTP treatment has demonstrated potential for enhancing the adhesive properties of dental silicate ceramics in laboratory experiments. Nonetheless, conventional HF etching remains superior to NTP in resin-ceramic bond strength and durability. The authors propose that subsequent studies should concentrate on atmospheric-pressure NTP devices utilising atmospheric gases for enhanced safety and practical application, particularly in dental repairs. The authors advocate testing the hypothesis concerning the effects of extended treatment durations or increased NTP power on surface characteristics using a three-dimensional regression model. Although the review indicates the availability of statistical models for data integration, it underscores a significant demand for multidirectional mathematical modelling to elucidate and optimise the intricate relationship between NTP parameters and their influence on ceramic surface attributes.

Awad et al. (2024) conducted an in-depth survey on NTP-aided ammonia (NH_3_) decomposition methods for the sustainable production of CO_x_-free hydrogen (H_2_). The study emphasises NH_3_’s role as a carbon-neutral H_2_ carrier and the advantages of integrating NTP with catalysis to address the drawbacks of conventional thermal processes. Conventional thermal catalysis for NH_3_ decomposition faces challenges such as high costs, limited noble metal availability for electrodes, and elevated reaction temperatures. NTP systems are preferred for NH_3_ decomposition due to their superior energy efficiency relative to thermal plasma systems. The study identified reactor and electrode configurations, power supply, NH_3_ flow rate and concentration, and catalyst as critical factors for optimising NTP-based NH_3_ degradation.

Komarzyniec et al. (2024) surveyed the non-linear electrical parameters and their influence on voltage and pulse generation in the NTP system design. They recommended that effective modelling of non-linear phenomena in plasma reactors can enhance operational efficiency and discharge uniformity. For example, increased power supply frequency can reduce high voltage in DBD discharge elements and improve GAD (gliding arc discharge) ignition efficiency. Innovations such as PPS (pulsed power supplies) with push–pull topology offer unique solutions for μGAD (micro-gliding arc discharge) supply. The topology’s limitations can act as benefits for μGAD generation, especially in air plasma conditions. This eliminates the necessity for supplementary ignition sources while enabling substantial voltage increases. They strongly emphasised the importance of analysing and modelling ferroresonance phenomena to alleviate negative impacts in industrial design (Komarzyniec et al., 2024).

The review by Wei et al. (2024) discussed two key frameworks for plasma modelling: MHD for thermal plasmas and Boltzmann’s equation for non-thermal plasmas. These frameworks serve as a solid basis for exploring plasma properties and their assumptions. Simulation of the electrical parameters of the NTP system under atmospheric pressure is essential for enhancing plasma processing performance. Despite progress, atmospheric plasma processing simulation remains in early development, facing significant challenges in achieving accurate diagnostics and automated control. Additionally, the modelling of atmospheric non-thermal plasma generation is still undeveloped, requiring further investigation into plasma kinetics and experimental validations. The survey also addressed the issues related to chemical equilibrium and modelling attempts of process–factor interactions in the complex non-thermal plasma environment.

In a review article, Machmud and Chang et al. (2023) and Hua et al. (2022) evaluated the degradation methods for gaseous fluorinated compounds (FCs) and found NTP techniques superior to TP in terms of activity, durability, and energy efficiency. The authors emphasised the necessity of computational modelling and simulations to enhance understanding and predict optimal conditions for plasma catalytic reactions. System reaction simulations specified that the interaction of FCs with H_2_ markedly improved the decomposition rate. Nonetheless, the simulation did not account for solid-state carbon formation. The reaction model outcomes revealed that FCs began to decompose at 285 K, leading to HF formation from FCs and H_2_ re-association. Low operating temperatures allowed for the detection of various products, including HF, H_2_O(g), CO, and CO_2_. From these simulations, it was concluded that incorporating H_2_ and O_2_ enhances FC decomposition in NTP environments. A two-step simulation process was recommended to investigate ionised field distribution and its influence on FC decomposition using DCPTUN, a specialised MHD (magnetohydrodynamic equations) numerical code for plasma simulation in ANSYS software (Choi et al., 2011; Hur et al., 2001).

Ye et al. (2022) presented a systematic review of catalyst modification advancements via NTP, including process conditions, reaction mechanisms, and real-life applications. It identifies NTP as a viable method for developing catalysts with innovative attributes due to its capacity to trigger chemical reactions at low temperatures and its environmentally friendly characteristics. The study extensively covers catalyst property modification, molecular-level changes, and surface chemistry improvements of NTP-modified catalysts and regeneration possibilities. The authors anticipate that the amalgamation of numerical methodologies with experimental validation and innovative characterisation techniques will be crucial for advancing the field and establishing novel paradigms in catalyst synthesis and materials engineering, yet specifics regarding modelling and simulations were not disclosed.

Fu et al. (2022) and Hertwig et al. (2018) reviewed the non-thermal plasma technology for microbial disinfection, covering its principles, active species mechanisms, and applications. The articles addressed the role of modelling and simulations in elucidating non-thermal plasma processes and optimising designs, especially for catalyst-assisted applications. The proposed EEDF (electron energy distribution function) and KDF (kinetic distribution function) approaches are significant to model active species formation and distribution from electron collisions during treatment. Furthermore, a 2D fluid model can simulate microplasma discharge phenomena to illustrate electron density in reaction zones. These computational strategies are essential for enhancing plasma-system design applicable for virus (Bumajdad and Khan, 2021) and other microbial disinfection.

However, according to the literature, the NTP treatment has been identified as superior to both thermal pyrolysis, thermal plasma (TP), and catalytic methodologies in terms of techno-economic efficiency, particularly with respect to the production of gaseous and liquid fuels from waste polyolefins (Song et al., 2024). For instance, the implementation of NTP treatment demonstrates considerable advantages when associating with conventional and catalytic pyrolysis in the generation of hydrogen, which stands as a sustainable and promising energy source derived from waste polyethylene. Although conventional pyrolysis is recognised as an established methodology, it is hindered by considerable energy demands and reduced effectiveness in hydrogen production.

**FIGURE 13:**
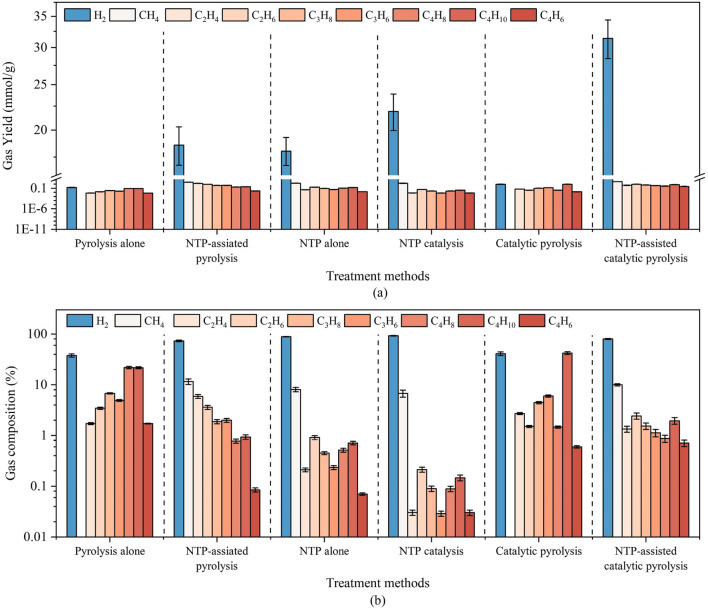
Quantification of gaseous output **(a)** and detailed analysis of the various components constituting the gas mixture **(b)** under a multitude of distinct treatment methodologies implemented to determine the resultant effects. Reproduced with consent from Elsevier.

Figure 13 distinctly illustrates that the incorporation of plasma within the pyrolysis system significantly enhances the yields of gaseous products. In terms of gas product outputs, sole pyrolysis yielded only minimal quantities of gas constituents, each falling below 0.15 mmol/g. Conversely, with the implementation of NTP-assisted pyrolysis, the yield of H_2_ increased to 18.56 mmol/g, and methane (CH_4_) reached 2.93 mmol/g. Regarding hydrogen selectivity, pyrolysis conducted in isolation exhibited a mere 37.81% H_2_ selectivity, whereas NTP-assisted pyrolysis increased this figure to 73.26%, with NTP alone achieving an even more impressive 88.83%. This suggests a pronounced preference for NTP for H_2_ formation during the reaction. Concerning the impact of catalytic performance, the introduction of a catalyst into the thermal pyrolysis system resulted in gas constituents for H_2_ and CH_4_ being less than 0.15 mmol/g; however, when a catalyst was integrated into the NTP system, the volumes of gas components surged to 31.41 mmol/g of H_2_ and 3.91 mmol/g of CH_4_. The H_2_ selectivity within the NTP system, combined with a catalyst, increased to 92.62%, while a solely catalytic system achieved approximately 88.0%. This underscores a synergistic effect whereby both NTP and the catalyst contribute positively to H_2_ selectivity. Furthermore, in the context of TP and catalytic processing, the plastic treatment necessitates an elevation of system temperature to between 750 °C and 1,000 °C for effective H_2_ production, whereas NTP processing can achieve analogous results at a significantly lower temperature of 250 °C. Thus, while TP and traditional thermal pyrolysis for hydrogen generation from polyolefins operate at elevated temperatures with accompanying disadvantages, non-thermal plasma provides a low-temperature, energy-efficient alternative. NTP markedly amplifies hydrogen yield and selectivity, particularly when amalgamated with catalytic pyrolysis, owing to its ability to generate active species and foster plasma–catalyst interactions. This positions NTP-assisted catalytic pyrolysis as an exceptionally promising technology for the sustainable production of hydrogen from plastic waste (Pandey et al., 2023; Song et al., 2024).

Overall, the insights derived from the comprehensive reviews of the existing literature underscore the varied and expanding applications of NTP technology across numerous domains, including environmental remediation, materials science, energy generation, water purification, microbial disinfection, food processing, and dentistry. It continually stresses the crucial importance of computational modelling, simulation, and advanced diagnostics in analysing, improving, and evolving NTP systems, whilst simultaneously acknowledging the present challenges and prospective research routes in each area.

All the aforementioned studies highlighted the significance of modelling and simulating the non-thermal plasma system and reaction nonlinearities; however, they markedly overlooked the requisite foundational knowledge in the realm of computational modelling and simulations. Additionally, a notable research void in the review efforts is the lack of an analysis focused on the reengineering of polyolefin-wastes.

## 5 Conclusion and future directions

The remarkable progress in computer hardware engineering, leading to enhanced memory and high-performance computing capabilities, has made it possible to solve nonlinear fluid dynamics equations in intricate electrochemical environments. This includes energy balance, heat, momentum, and mass transfer through various numerical methods in electrochemical process engineering. These advancements have spurred the development of more practical numerical approaches, resulting in a combination of multiple computational techniques and codes. In this mini-review, scientific efforts in advancing modelling and simulation for NTP-assisted conversion of “discarded polyolefins” (commonly known as waste plastics) are highlighted as a promising emerging technology with great potential for mitigating pollution and combating global warming. The careful selection of models and the decision-making process in choosing the right set of equations to understand complex electrochemical reaction dynamics and system behaviours are crucial for the design and scale-up of NTP systems.

There remain several key issues and challenges in the field of NTP process engineering that must be addressed: (1) The NTP modification process is highly complex, combining multiple disciplines such as thermodynamics, physics, chemistry, and materials science. Unfortunately, there is still a dearth of *in situ* computational platforms and integrated approaches, making it challenging to quickly analyse the modification procedure. Future studies should focus on developing and adapting numerical methods for real-time, *in situ* analysis of both NTP and TP processes with high temporal and spatial resolution. (2) Key parameters such as input energy, carrier gas, reactor configuration, and reaction time significantly influence the modification process. To fully implement NTP treatment, these factors need to be thoroughly investigated and analysed. Additionally, designing a plasma reactor that ensures a uniform and stable discharge, based on simulation predictions, is critical. At present, the “trial-and-error” approach dominates, which hinders progress and necessitates more focused research. (3) The majority of NTP-modified experiments remain at the laboratory scale. More effort should be devoted to overcoming the barriers to scaling up and applying these processes in industrial settings to enhance the commercial viability of NTP reactors and their integration with energy supply grids.

The advanced models and simulations developed for NTP conversion of solid plastic waste into various gaseous and liquid fuels across different scales offer exciting possibilities for other areas in electrochemical engineering. As such, integrating modern engineering trends, such as artificial intelligence (AI) and machine learning (ML), into “real-world” NTP applications in the waste-to-energy sector warrants further investigation. Achieving this ambitious goal requires diversifying numerical methods or models capable of predicting the necessary real-time plasma conditions. Only by combining numerical approaches with experimental validation and innovative data acquisition techniques can the “black box” mechanism of plasma be uncovered, establishing a new standard in chemical synthesis and electrical engineering.

## Nomenclature

**0D/1D**: Zero-dimensional/one-dimensional
**2D**: Two-dimensional
**3D**: Three-dimensional
**C**: Carbon
**CFD**: Computational fluid dynamics
**CGE**: Cold gas efficacy
**CO**_**2**_: Carbon dioxide
**DBD**: Dielectric barrier discharge
**EEDF**: Electron energy distribution function
**EIA**: Environmental impact assessment
**EoS**: Equation of state
**eqn**: Equation
**ER**: Equivalence ratio
**GHGs**: Greenhouse gases
**KDF**: Kinetic distribution function
**LJ**: Lennard–Jones (intermolecular potential)
**MHD**: Magnetohydrodynamic equations
MHz: Megahertz
**MJ/kg**: Mega joules per kilogram
**NOx**: Nitrogen oxides
**NTP**: Non-thermal plasma
**PAV**: Plasma-assisted valorisation
**SAFT**: Statistical associating fluid theory
**SEI**: Specific energy input (J cm^-3^ or kj/L)
**SFR**: Steam-to-fuel ratio
**soft-SAFT**: A variant of the SAFT equation
**SOx**: Sulphur oxides
**TP**: Thermal plasma
**Wplasma-NTP**: Plasma power consumption per mass flow rate of waste (kwh/tonne-waste)

### List of symbols

***(r, θ, z)***: Fluids flow in directions in the cylindrical coordinate system
${\vec{u}}_{m}$: Mixture velocity
$\vec{u_{l}}$: Velocity of phase-1
$a_{l}$: Volume fraction
${k\;\Gamma }_{m}^{k}$: Mixture diffusivity for the scalar
$m_{e}$: Electron mass
$m_{i}$: Ion mass
$S_{\phi_{k}\;}$: Source term for scalar k in phase
$\Gamma_{k}$: Diffusion coefficient
$\Gamma_{k}I$: Diffusion coefficient for scalar k in phase-1
$\rho_{m}$: Mixture density
$\phi_{k}$: Arbitrary scalar variable
$\phi^{k}$: Scalar of mixture of phases
$\phi_{l}^{k}$: Arbitrary scalar k in phase
∂/∂z: Partial derivative with respect to the axial direction (z)
***∂/∂θ***: Partial derivative with respect to the angular direction (θ)
***µs***: Microseconds
***A***_***s***_: Substrate cross-sectional area
***C***_***d***_: Capacitance of the dielectric
***C***_***D***_: Effective capacitance of the feedstock
***C***_***f***_: Capacitance of the feedstock
***D***: Data-fitting constant
$d\vec{S}$: Face vector area
***E***: Exergy flow
***Ėx***: Exergy flows
***Ėx***_***in***_: Exergy inflow
***Ėx***_***out***_: Exergy outflow
***e***_***ch***_: Elementary charge
***E***_***i***_: Excitation energy
***E***_***ion***_: Ionisation energy of the gas
***ex***_***ch***_: A chemical exergy
***F***: Convective flux
***fY***: Unconditional density function
***fy|xi***: Conditional density function
***h***: Enthalpy
***h***_***0***_: Standard enthalpy
***I***: Isotropic diffusivity
***I***_***ij***_: Relative intensity of the spectral emission
***I***_***p***_ ***(t)***: Plasma current
***I***_***p,g***_ ***(t)***: Displacement current through the gap
***I***_***t***_ ***(t)***: Total input current
***k***_***B***_: Boltzmann constant
***m˙***: Mass flow
***mmol/g***: Millimoles per gram
***ne***: Electron number density
***Q˙***: Heat transfer rate
***r***: Radial direction in the cylindrical coordinate system.
***s***: Entropy
***S***_***ϕk***_: Source term for each N scalar equation
***s***_***0***_: Standard entropy
***T***_***exc***_: Excitation temperature in electron volts (ev)
***U***_***a***_ ***(t)***: Total input voltage
***U***_***D***_ ***(0)***: Memory voltage
***U***_***D***_ ***(t)***: Effective dielectric voltage
***U***_***d***_ ***(t)***: Voltage across the dielectric
***U***_***f***_ ***(t)***: Feedstock voltage
***U***_***p***_ ***(t)***: Plasma voltage
***V***: Volts
***Vsh***: Sheath potential
$\dot{W}$: Work rate
***x***_***i***_: Molar fraction of gas species
***z***: Axial direction in the cylindrical coordinate system
***δi***: Sensitivity indicator
***δi***: Average variations in fy|xi relative to fy
***ε***_***0***_: Permittivity of free space
***θ***: Angular direction in the cylindrical coordinate system
***κ***_***d***_: Dielectric constant of the dielectric
***κ***_***f***_: Dielectric constant of the feedstock
***λ***_***ij***_: Wavelength of the emitted spectra
***ρ***_***1***_: Physical density of phase
